# Excess Cardiovascular Burden Among Young Patients with Cancer: A Systematic Review and Meta-Analysis †

**DOI:** 10.3390/cancers18213579

**Published:** 2026-11-07

**Authors:** Musa Kiyani, Robert J. Heins, John Hayssam Yazji, Harith Almimar, Gilberto de Lima Lopes

**Affiliations:** 1 University of Miami/HCA Florida JFK Hospital, Atlantis, FL 33462, USA; harith.almimar@hcahealthcare.com; 2 College of Osteopathic Medicine, Kansas City University, Kansas City, MO 64106, USA; robert.heins@kansascity.edu (R.J.H.); john.yazji@kansascity.edu (J.H.Y.); 3 Sylvester Comprehensive Cancer Center, University of Miami, Miami, FL 33136, USA; glopes@med.miami.edu

**Keywords:** adolescent and young adult cancer, cardiovascular disease, cardiovascular mortality, cardio-oncology, survivorship, meta-analysis

## Abstract

**Background**: Young patients with cancer may develop cardiovascular complications decades earlier than the general population, yet their long-term cardiovascular risk remains incompletely defined. Most prior evidence derives from older adults or therapy-specific analyses, leaving adolescents and young adults understudied as a distinct population. **Methods**: We performed a systematic review and meta-analysis of observational cohort, registry-based, and population-based studies evaluating cardiovascular outcomes or mortality among young patients with cancer (adolescents and young adults [15–39 years], <45 years, <50 years, or clearly defined young age subgroups) compared with age-matched comparators. PubMed/MEDLINE, Embase, Scopus, and Web of Science were searched from inception through 26 February 2026. Two reviewers independently screened studies and assessed risk of bias using the Newcastle–Ottawa Scale. Evidence certainty was evaluated with GRADE. Random-effects models were used for pooled analyses. **Results**: Fifty-eight studies were included. Young patients with cancer had significantly increased risks of heart failure (HR, 4.08; 95% CI, 2.20–7.57), pulmonary embolism (HR, 4.63; 95% CI, 3.03–7.06), venous thrombosis (HR, 4.06; 95% CI, 2.51–6.58), and cardiomyopathy (HR, 3.52; 95% CI, 2.21–5.63). Cardiovascular mortality was also elevated (SMR, 4.30; 95% CI, 3.06–6.05), although a trim-and-fill analysis resulted in an adjusted pooled SMR of 1.79 (95% CI, 1.05–3.05, *p* = 0.0313). Overall cardiovascular disease risk was approximately 2-fold higher, with additional increases in stroke, arrhythmia, and hypertension. Heterogeneity was high for several key outcomes (I^2^, overall cardiovascular disease HR 96.0%; cardiovascular mortality SMR 99.1%; heart failure HR 92.5%; venous thrombosis HR 94.1%), and evidence certainty was low for most. **Conclusions**: Young patients with cancer face substantially increased risks of cardiovascular disease, particularly heart failure, cardiomyopathy, and thrombotic complications. Prospective studies are needed to determine whether earlier cardiovascular surveillance improves outcomes.

## Simple Summary

Young patients with cancer face substantially elevated risks of heart failure, cardiomyopathy, pulmonary embolism, and venous thrombosis, along with increased risks of stroke, arrhythmia, and cardiovascular mortality compared with age-matched populations. These findings identify cardiovascular disease as an important late effect in this population, though prospective evaluation is needed to determine whether earlier or intensified surveillance improves outcomes.

## 1. Introduction

Cardiovascular disease (CVD) has emerged as a leading cause of non-cancer morbidity and mortality among cancer survivors, with consistently elevated risks of cardiac death, heart failure, and atherosclerotic events compared with age-matched populations [1,2]. Advances in oncologic therapy have substantially improved survival, with 5-year survival among adolescents and young adults (AYA; ages 15–39 years) now approaching 86%, resulting in a rapidly expanding population of long-term survivors exposed to the late effects of cancer and its treatment [3]. Young patients with cancer also represent a distinct population in whom cardiovascular risk is both amplified and clinically consequential. Exposure to cardiotoxic therapies—including anthracyclines, chest radiation, and alkylating agents—occurs during a period of otherwise low baseline cardiovascular risk, with adverse effects often manifesting years to decades after treatment. When cardiovascular events occur, outcomes appear disproportionately severe relative to non-cancer patients [4].

Despite these concerns, the overall cardiovascular burden in young patients with cancer remains incompletely characterized. Prior studies have largely focused on older populations, single malignancies, or specific treatment-related toxicities, limiting generalizability across the full spectrum of cardiovascular disease. A recent meta-analysis synthesized cardiovascular risk across all-age cancer survivors and identified younger age as a moderator, but did not provide condition-specific estimates within the young-patient population or address cardiovascular mortality [5]. Although consensus surveillance recommendations exist, the optimal timing, intensity, and scope of cardiovascular monitoring in young patients with cancer remain uncertain [6]. Comprehensive population-level estimates spanning structural, thromboembolic, cerebrovascular, and arrhythmic outcomes in this population are lacking. To our knowledge, no prior meta-analysis has provided condition-specific cardiovascular risk estimates or quantified cause-specific cardiovascular mortality in childhood, adolescent, and young-adult cancer populations; this synthesis addresses both gaps.

The clinical implications of this evidence gap extend beyond survivorship research. Cardiovascular disease has become an increasingly important determinant of long-term health in young cancer survivors, frequently competing with recurrent malignancy as a cause of morbidity and mortality. Accurate quantification of cardiovascular risk across individual disease domains may help inform risk stratification, guide survivorship surveillance, and identify patient populations most likely to benefit from early preventive interventions. Additionally, as the population of young cancer survivors expands, healthcare systems are increasingly tasked with balancing effective cancer surveillance with long-term cardiovascular care. Accurate estimates of both absolute and relative cardiovascular risk are important to support evidence-based clinical guidelines, inform shared decision-making, and prioritize future research evaluating preventive strategies and survivorship interventions in this unique population.

In this systematic review and meta-analysis, we aimed to quantify the relative risk of cardiovascular disease and cardiovascular mortality across multiple disease domains in young patients with cancer compared with age-matched populations.

## 2. Materials and Methods

A systematic literature search was conducted in accordance with the Preferred Reporting Items for Systematic Reviews and Meta-Analyses (PRISMA) and the Meta-Analysis Of Observational Studies in Epidemiology (MOOSE) guidelines (Table A1) [7,8]. PubMed/MEDLINE, Embase, Scopus, and Web of Science were searched from database inception through 26 February 2026, using terms related to young patients with cancer and cardiovascular disease (Appendix B). The protocol was prospectively registered on the International Prospective Register of Systematic Reviews (PROSPERO) with the registration number CRD420261414899. The study selection process is summarized in a PRISMA diagram (Figure S1).

Eligible studies were English-language observational cohort, registry-based, population-based, or randomized analyses of young patients with cancer, defined as adolescents and young adults (AYA; 15–39 years), patients <45 years, patients <50 years, or other clearly defined young age subgroups as specified by individual studies, reporting incident cardiovascular outcomes after cancer diagnosis. Studies were required to include a comparator group (age-matched non-cancer individuals, general population references, sibling controls, or internal comparisons) or provide standardized incidence or mortality ratios, and to report quantitative outcomes with effect estimates (HR, RR, IRR, SMR, SIR) or data sufficient to derive them. We excluded studies restricted to older adults without extractable young-subgroup data, case reports, case series without comparators, reviews, editorials, conference abstracts, studies of subclinical markers only, and non-human studies.

Titles and abstracts were screened independently and in duplicate by 2 qualified reviewers (R.J.H. and J.H.Y.), followed by independent full-text review of potentially eligible studies. Discrepancies were resolved through discussion and adjudication by a third reviewer (M.K.).

Data were extracted independently using standardized forms in Google Sheets (Google LLC, Mountain View, CA, USA; https://docs.google.com/spreadsheets; accessed on 1 June 2026). Extracted variables included study design, country, sample size, sex, cancer type, age group definition, follow-up duration, comparator characteristics, and outcome definitions. Cardiovascular outcomes extracted included angina or chest pain, overall cardiac conditions, overall cardiovascular disease, circulatory system disease, cardiac arrhythmia, ischemic heart disease, coronary artery disease, myocardial infarction, cardiomyopathy, heart failure, valvular heart disease, pericardial disease, cerebrovascular disease, stroke, hypertension, pulmonary embolism, and venous thrombosis. Mortality outcomes included mortality from: overall cardiovascular disease, circulatory system disease, cardiac ischemia, cardiomyopathy, valvular heart disease, and cerebrovascular. Effect estimates were extracted as reported by individual studies, including HR, RR, IRR, SMR, SIR, and SHR, along with corresponding 95% confidence intervals and *p*-values. Where multiple effect estimate types were reported for the same outcome, each was extracted and pooled separately. Where multiple publications drew from the same underlying cohort but reported different cardiovascular outcomes or disease-specific subsets, each publication was retained for its respective outcome. Patients may therefore contribute to more than one outcome estimate. We report “person-outcomes,” defined as the sum of participants across all studies contributing to a given outcome, allowing for overlap, rather than unique participant counts, because standard participant totals would inflate the apparent number of unique individuals contributing to each outcome.

Risk of bias was assessed independently by 2 reviewers (R.J.H. and J.H.Y.) using the Newcastle–Ottawa Scale (NOS) for observational studies (Table S1) [9]. We used GRADE (Grading of Recommendations Assessment, Development and Evaluation) to evaluate the quality of evidence for each pooled analysis (Table S2) [10].

Statistical analyses were conducted using the meta package in R (version 4.5.1; R Foundation for Statistical Computing, Vienna, Austria) within RStudio (version 2025.05.1+513; Posit Software, PBC, Boston, MA, USA). RStudio is available at https://posit.co/products/open-source/rstudio/ (accessed 4 June 2026). A random-effects model accounted for between-study heterogeneity. The between-study variance (τ^2^) was estimated using the Restricted Maximum Likelihood (REML) method, and confidence intervals were calculated using the Hartung–Knapp adjustment. A minimum of 3 studies was required for pooling of effect estimates; outcomes reported by ≤2 studies were not eligible for meta-analysis and are instead presented descriptively. SMRs were pooled using a generic inverse-variance random-effects model on the log-transformed SMR, an accepted approach when only summary effect estimates and corresponding confidence intervals are available. Included studies did not provide sufficient data (e.g., observed and expected deaths) to implement Poisson-based or Generalized Linear Mixed Models (GLMMs) approaches.

A *p*-value of less than 0.05 was the threshold for statistical significance. Heterogeneity was assessed using the I^2^ statistic, with I^2^ greater than 75% considered indicative of substantial heterogeneity. With substantial heterogeneity and at least 10 studies available, meta-regression analysis identified potential sources of variability. A 95% prediction interval (PI) calculation was carried out for outcomes with >75% heterogeneity and ≥5 included studies. For publication bias, Egger’s test was used when at least 10 studies were available during pooling. Following confirmation of significant publication bias via Egger’s test, trim-and-fill analysis was conducted to estimate the adjusted effect size in the absence of publication bias.

## 3. Results

### 3.1. Study Selection and Characteristics

Fifty-eight studies drawing from 36 unique cohort sources were included. Study characteristics are summarized in Tables S3 and S4, and the cohort sources contributing more than one publication are summarized in Table S5. Certainty of evidence ranged from very low to moderate across pooled outcomes (Table S2). Heterogeneity was high for several key pooled outcomes, including overall cardiovascular disease (HR I^2^ 96.0%), heart failure (HR I^2^ 92.5%), venous thrombosis (HR I^2^ 94.1%), and cardiovascular mortality (SMR I^2^ 99.1%) (Table 1). Effect estimates were pooled for 13 cardiovascular condition outcomes and five mortality outcomes. Per-outcome study counts and person-outcome totals are reported in Table S6. Outcomes with fewer than three studies were not pooled and are summarized in Table S7 [11,12,13,14,15,16].

### 3.2. Cardiovascular Conditions

#### 3.2.1. Overall Cardiovascular Disease

**Figure 1:**
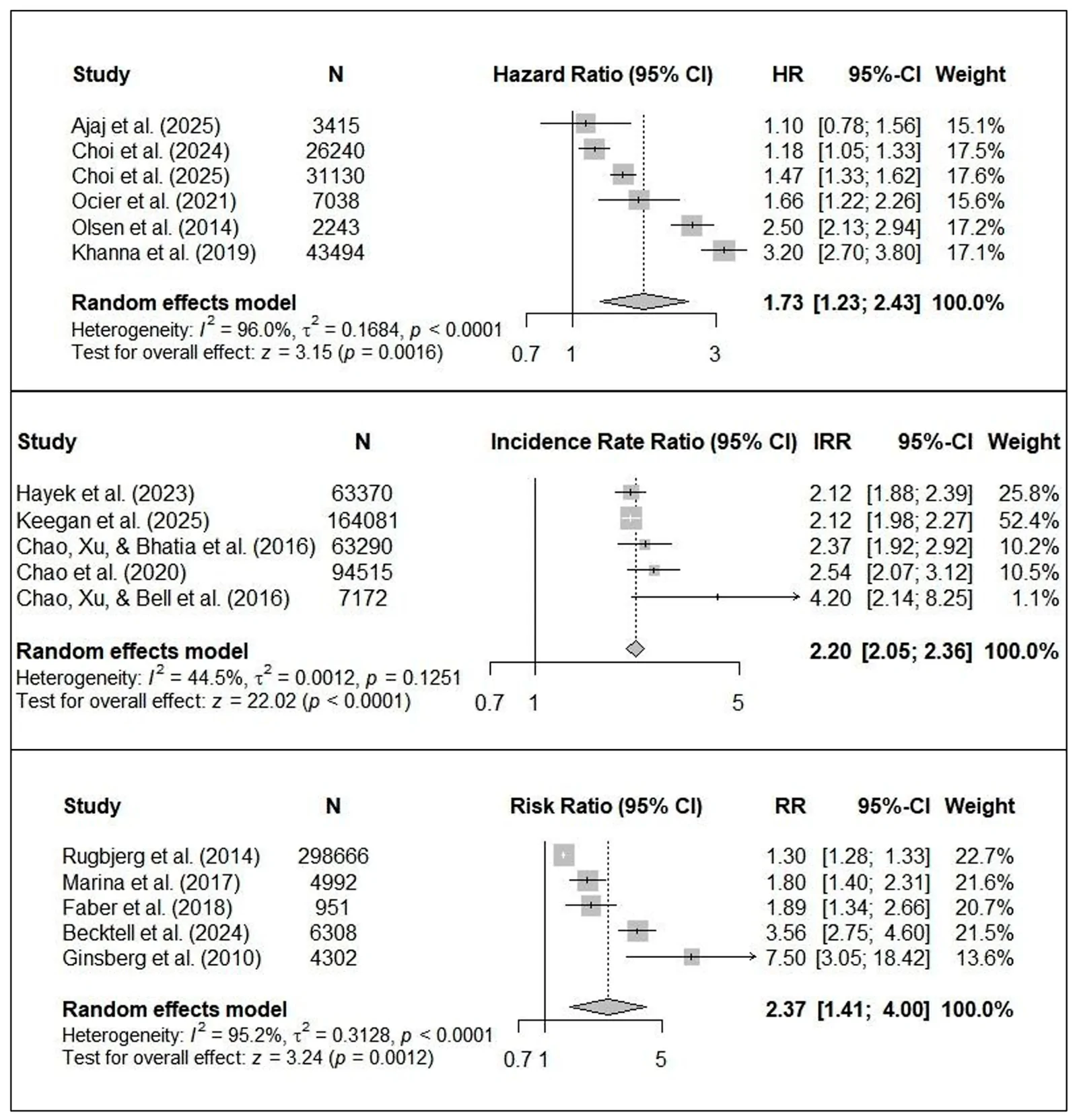
Forest plots of overall cardiovascular disease risk in young patients with cancer, stratified by effect measure: hazard ratio (**top**), incidence rate ratio (**middle**), and risk ratio (**bottom**). All analyses used a random-effects model. N reflects the total number of participants per study [17,18,19,20,21,22,23,24,25,26,27,28,29,30,31,32]. CI, confidence interval; HR, hazard ratio; IRR, incidence rate ratio; RR, risk ratio.

Sixteen studies reported overall cardiovascular disease, encompassing 821,207 person-outcomes [17,18,19,20,21,22,23,24,25,26,27,28,29,30,31,32]. Cancer was associated with a significantly increased risk of cardiovascular disease across all effect measure types (Table 1 and Figure 1), including as hazard ratios (HR 1.73, 95% CI 1.23–2.43, 95% PI 0.55–5.42; six studies), incidence rate ratios (IRR 2.20, 95% CI 2.05–2.36; five studies), and risk ratios (RR 2.37, 95% CI 1.41–4.00, 95% PI 0.43–13.26; five studies). Although the pooled HR and RR demonstrate significant increase in cardiovascular risk, the corresponding prediction intervals are wide and include the null value. This indicates substantial between-study heterogeneity, and suggests that the magnitude of this association may vary considerably across comparable cancer populations.

#### 3.2.2. Cardiomyopathy and Heart Failure

Heart failure was reported in 14 studies with 471,215 person-outcomes [17,22,23,24,28,29,30,31,32,33,34,35,36,37], and demonstrated a significantly elevated risk among those with cancer (HR 4.08, 95% CI 2.20–7.57, 95% PI, 0.48–34.79; nine studies) (Figure S2). The 95% prediction interval for the HR is wide and crossed the null. This indicates that the true effect in a new, comparable study could range from a modest protective association to a very large increase in risk.

Cardiomyopathy was reported in nine studies with 478,405 person-outcomes [19,23,26,27,30,31,33,38,39], with a significantly elevated risk among those with cancer (HR 3.52, 95% CI 2.21–5.63, 95% PI 0.82–15.17; six studies; IRR 3.38, 95% CI 2.68–4.26; three studies) (Figure S3). Note that the wide PI for the HR estimate limits the confidence of this finding, by showing that the true HR in a new, comparable study could range from a slightly protective association to a more than 15-fold increase in risk. Despite the statistically significant pooled HR, there appears to be substantial variation in the magnitude of the cancer–cardiomyopathy association across studies, and the pooled estimate should be interpreted with caution. A prediction interval was not calculated for the IRR estimate given its comparatively lower heterogeneity (I^2^ = 65.0%).

#### 3.2.3. Cardiac Arrhythmia

Cardiac arrhythmia was assessed in 13 studies with 692,772 person-outcomes [17,22,23,25,28,29,30,31,32,35,38,39,40]. Having cancer was significantly associated with an increased risk of arrhythmia on analysis by hazard ratio (HR 1.37, 95% CI 1.19–1.58; eight studies), though this association did not reach significance when pooled by risk ratio (RR 1.48, 95% CI 0.98–2.25 95% PI 0.40–5.46; five studies), with substantial heterogeneity across studies (I^2^ 81.9%) (Figure S4). Values of both the CI and the PI indicated that the pooled estimate is statistically imprecise and that individual studies vary widely in the direction and magnitude of association.

#### 3.2.4. Ischemic Heart Disease and Myocardial Infarction

Ischemic heart disease was assessed in four studies with 100,200 person-outcomes [22,23,31,39]. The pooled hazard ratio did not reach statistical significance (HR 1.66, 95% CI 0.82–3.37), with considerable heterogeneity across studies (I^2^ 92.1%) (Figure S5).

Myocardial infarction was assessed in six studies with 154,483 person-outcomes [17,22,23,33,34,38]. The pooled hazard ratio did not demonstrate statistical significance (HR 1.40, 95% CI 0.69–2.86), with moderate heterogeneity (I^2^ 70.5%) (Figure S6).

#### 3.2.5. Valvular Heart Disease and Pericardial Disease

Valvular disease was reported in five studies with 102,162 person-outcomes [23,28,30,31,34]. Cancer was significantly associated with an elevated risk of valvular heart disease (HR 2.41, 95% CI 1.31–4.46, 95% PI 0.36–16.31) (Figure S7 in Supplement S1). However, the wide range of the PI indicates that the true effect in a new, comparable study could range from a protective association to a more than 16-fold increase in risk. This reflects substantial inconsistency across studies. The statistically significant pooled estimate should be interpreted with caution. Pericardial disease was assessed in four studies with 67,409 person-outcomes [17,28,30,34], and was similarly elevated in cancer patients (HR 2.41, 95% CI 1.24–4.68) (Figure S8).

#### 3.2.6. Stroke and Cerebrovascular Disease

Stroke was evaluated in 11 studies with 609,822 person-outcomes [17,19,22,23,26,27,31,33,35,40,41]. Having cancer was associated with a significant risk of stroke (HR 1.33, 95% CI 1.10–1.61) (Figure S9).

Cerebrovascular disease was assessed in four studies with 102,312 person-outcomes [22,23,39,42]. The pooled hazard ratio did not reach statistical significance (HR 2.13, 95% CI 0.92–4.92), and demonstrated high heterogeneity (I^2^ 91.5%) (Figure S10).

#### 3.2.7. Pulmonary Embolism and Venous Thrombosis

Pulmonary embolism was assessed in three studies with 75,559 person-outcomes [22,23,33] and venous thrombosis in five studies with 135,054 person-outcomes [22,23,31,33,38]. Cancer was associated with the largest cardiovascular effect sizes observed across all conditions, with a markedly elevated risk of both pulmonary embolism (HR 4.63, 95% CI 3.03–7.06) (Figure S11) and venous thrombosis (HR 4.06, 95% CI 2.51–6.58) (Figure S12).

#### 3.2.8. Hypertension

Hypertension was assessed in seven studies with 473,141 person-outcomes [19,20,26,29,32,35,43]. Cancer was significantly associated with an increased risk of hypertension (IRR 1.34, 95% CI 1.25–1.44; three studies and RR 1.29, 95% CI 1.10–1.52; four studies) (Figure S13).

### 3.3. Cardiovascular Mortality

#### 3.3.1. Overall Cardiovascular Mortality

**Figure 2:**
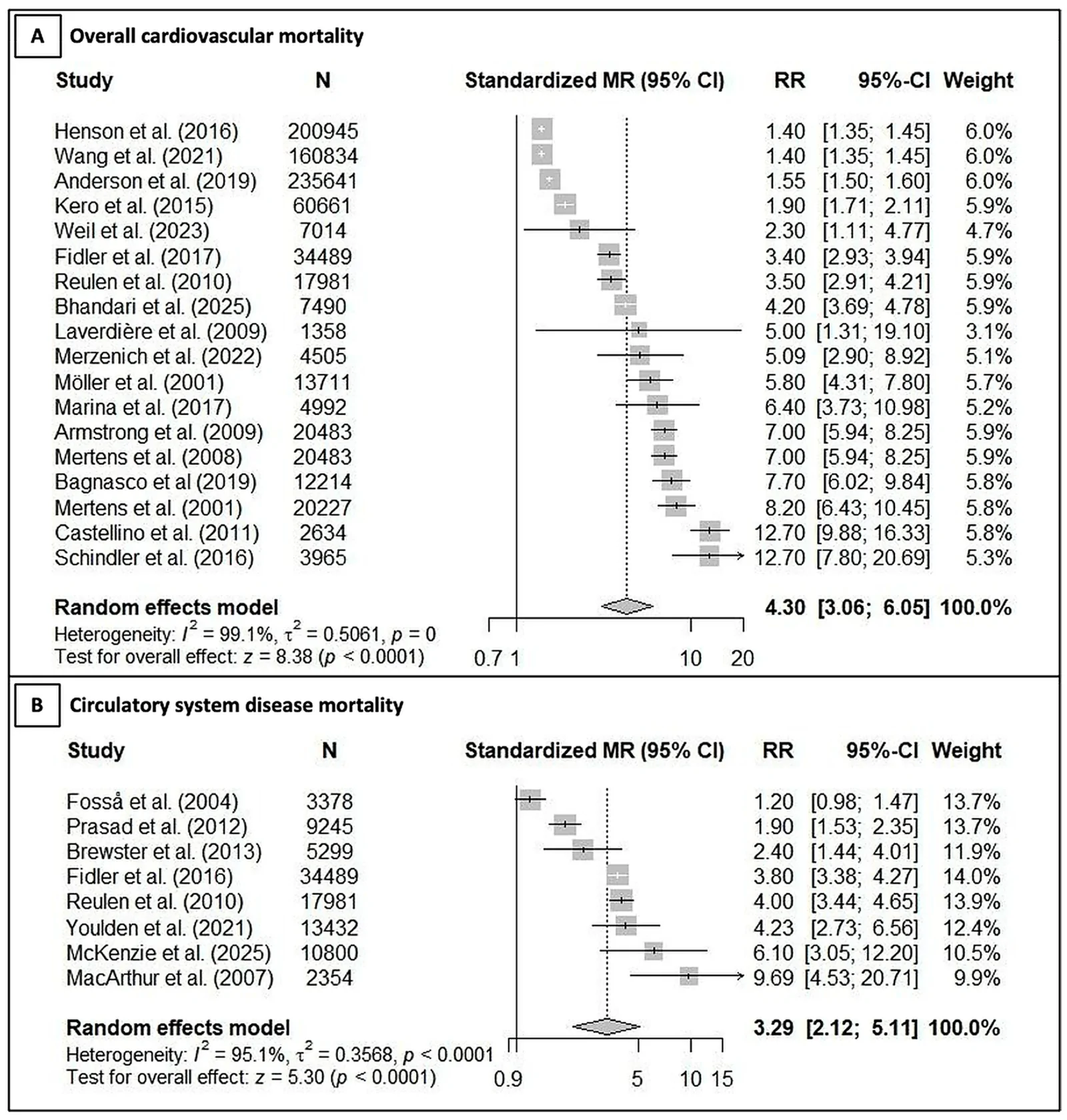
Forest plots of cardiovascular mortality in young patients with cancer: (**A**) overall cardiovascular mortality and (**B**) circulatory system disease mortality, reported as standardized mortality ratios [1,2,29,37,44,45,46,47,48,49,50,51,52,53,54,55,56,57,58,59,60,61,62,63,64]. All analyses used a random-effects model. N reflects the total number of participants per study. CI, confidence interval; MR, mortality ratio; RR, risk ratio.

Cardiovascular mortality was reported in 18 studies with 829,627 person-outcomes [1,2,29,37,44,45,46,47,48,49,50,51,52,53,54,55,56,57]. Compared to the general population, young patients with cancer had a significantly higher risk of cardiovascular death (SMR 4.30, 95% CI 3.06–6.05, 95% PI 0.92–20.16) (Figure 2). Again, note that the PI was wide and included the null value (SMR = 1), indicating substantial between-study heterogeneity. This suggests that although the pooled standardized mortality ratio indicates a 4-fold risk in cardiovascular mortality among cancer patients, the magnitude of this association may vary markedly across comparable populations. These findings demonstrate high confidence that an overall association exists, but lower confidence in the exact effect size and its consistency across populations and settings.

Circulatory system disease mortality was reported in eight studies with 96,978 person-outcomes [56,58,59,60,61,62,63,64]. Cancer was similarly associated with a significantly elevated risk of death (SMR 3.29, 95% CI 2.12–5.11, 95% PI 0.73–14.87). Despite the statistical significance of this association, the wideness of the PIs suggest that the magnitude of this effect may vary across different cancer populations.

#### 3.3.2. Cause-Specific Cardiovascular Mortality

Cardiac ischemia mortality was reported in four studies with 298,729 person-outcomes [1,48,49,50]. The pooled estimate was elevated (SMR 3.15, 95% CI 1.05–9.47) but should be interpreted with caution given the wide confidence interval with a lower bound near unity and substantial heterogeneity across contributing studies (I^2^ 98.7%).

Cardiomyopathy mortality was reported in three studies with 238,068 person-outcomes [1,48,49]. The pooled estimate did not reach statistical significance (SMR 8.10, 95% CI 0.97–67.89), with extremely high heterogeneity precluding reliable interpretation of this estimate (I^2^ 99.2%).

Cerebrovascular disease mortality was reported in four studies with 417,090 person-outcomes [2,44,48,56]. Cancer was associated with a significantly increased risk of death from cerebrovascular disease (SMR 2.44, 95% CI 1.17–5.09), though heterogeneity across studies was high (I^2^ 96.7%).

### 3.4. Publication Bias, Sensitivity Analysis, Meta-Regression

Formal assessment of publication bias using Egger’s test was limited by the number of studies available per outcome and could be performed for only one outcome. Egger’s test detected significant evidence of publication bias for overall cardiovascular mortality (bias estimate 11.20, SE 1.97, *p* < 0.0001). Trim-and-fill analysis imputed nine hypothetical missing studies on the left side, yielding an adjusted pooled SMR of 1.79 (95% CI, 1.05–3.05, *p* = 0.0313) versus an unadjusted estimate of 4.30 (95% CI 3.06–6.05, *p* < 0.0001). However, this outcome showed substantial heterogeneity (I^2^ = 99.1%), which limits interpretability of these results. Funnel plot asymmetry under high heterogeneity may reflect genuine variability in true effects across studies rather than publication bias. The trim-and-fill-adjusted estimate should therefore not be interpreted as a corrected true effect. The discrepancy between adjusted and unadjusted estimates more likely reflects underlying heterogeneity than publication bias alone. Further, demographic and methodological diversity across studies should be considered the primary explanation for the funnel asymmetry, and this outcome should be interpreted with substantial caution.

We performed sensitivity analysis using the leave-one-out approach. There was no change in the directional effect of the estimate or the significance expressed by the *p*-value for any of the outcomes. Only one study had a NOS ≤ 5 [43]. It was used for the outcomes of hypertension (with RR as the effect estimate). Its exclusion from the RR analysis changed the effect estimate from RR = 1.29 (95% CI, 1.10–1.52, *p* = 0.0019) to RR = 1.20 (95% CI, 1.1585–1.2473, *p* < 0.0001). There was no meaningful change either in the directional effect of the RR or its *p*-value and hence the study was kept in the analysis.

We included 58 studies drawn from only 36 unique cohort sources, raising the possibility of overlap, whereby the same participants may have contributed to the same outcome more than once. To address this, we conducted a sensitivity analysis restricted to one study per cohort per outcome. When a cohort contributed more than one eligible study, we selected the study with the larger sample size or longer follow-up duration as the tiebreaker. We defined a significant change as a change in the significance of the *p*-value or in the direction of effect. Of the eight outcomes for which this adjustment was made, none showed a significant change. This is shown in Table S8.

Meta-regression was performed for overall cardiovascular mortality and tested country and percentage of male participants as moderators (Table S9). Other potential moderators, including age range, time since diagnosis, follow-up duration, cancer type, and treatment exposure, were not analyzed because data were either insufficiently reported across studies or categories were not sufficiently distinct. A higher proportion of male participants was associated with a greater risk of cardiovascular death. No country was significantly associated with cardiovascular mortality.

## 4. Discussion

Our meta-analysis provides condition-specific cardiovascular risk estimates and cause-specific mortality data in an AYA-specific population, extending beyond the composite outcome and all-age scope of the largest prior synthesis, Li et al. [5]. Heart failure and cardiomyopathy emerged as dominant cardiovascular signals, while cardiovascular mortality was also significantly elevated. Risk was consistently elevated across structural, cerebrovascular, arrhythmic, and thromboembolic domains. Substantial heterogeneity across outcomes reflects both the clinical diversity of this population and methodological variability across studies. Outcomes with the highest heterogeneity (overall cardiovascular disease HR, heart failure HR, venous thrombosis HR, and cardiovascular mortality SMR, each I^2^ > 90%) should be interpreted as reflecting a consistent direction of elevated risk rather than a precise, generalizable magnitude, given the substantial variation in cancer type, age range, follow-up duration, and treatment era across contributing studies.

Beyond the statistical heterogeneity reported for individual pooled outcomes, the clinical heterogeneity of the pooled population itself warrants explicit consideration. Included studies spanned childhood cancer survivors, adolescent and young adult populations, and young adults up to age 50 years, as well as both hematologic and solid tumor diagnoses across treatment eras spanning several decades. These subgroups are not clinically interchangeable: AYA cancer survivors have been shown to carry a significantly higher cardiovascular disease risk than childhood cancer survivors [65], and age at cancer diagnosis has itself been identified as a critical determinant of cardiac mortality risk, with fold-elevation varying substantially by primary cancer diagnosis [49]. Combining these subgroups into a single pooled estimate may therefore obscure clinically meaningful differences in risk magnitude, and pooled estimates in this analysis should be interpreted as reflecting a composite signal across a heterogeneous population rather than a risk estimate generalizable to any single age or diagnostic subgroup. Age category, cancer type, and treatment era subgroup analyses were precluded by insufficient granularity in study-level reporting, as detailed in Limitations, underscoring the need for future studies with sufficient stratified data to clarify risk within more narrowly defined populations.

The convergence of elevated structural risk with increased cardiovascular mortality suggests a contribution from cancer-directed therapy; however, because our exposure definition combines cancer diagnosis and treatment exposure, and treatment-stratified data were unavailable, the relative contribution of malignancy itself versus treatment-related cardiotoxicity cannot be determined from this analysis [66]. Valvular and pericardial disease produced parallel, lower-magnitude patterns, and cause-specific mortality from cardiac ischemia and cerebrovascular disease reinforced the overall mortality signal. Meta-regression identified male sex as a moderator of cardiovascular mortality, consistent with established sex differences potentially reflecting treatment exposure, comorbidity burden, and survivorship care [67].

Thromboembolic disease showed markedly elevated risk, likely reflecting cancer-associated hypercoagulability, central venous access, and immobilization [68]. Although less emphasized than structural cardiotoxicity in cardio-oncology guidance, this magnitude suggests thromboembolic disease warrants further evaluation as a component of cardiovascular risk in this population, pending prospective data to inform surveillance recommendations. Atherosclerotic and cerebrovascular signals were more discordant: stroke was elevated; whereas, myocardial infarction and ischemic heart disease were not, likely reflecting latency, ascertainment differences, and competing risks in younger populations. Arrhythmia was elevated by HR but not RR, likely reflecting time-at-risk structure rather than absence of association. Hypertension was elevated, consistent with treatment-related contributions from steroids, nephrotoxins, and radiation [69]. These relative risk estimates should be interpreted alongside baseline cardiovascular event rates in young populations, which are low; absolute risk differences may therefore be more modest than relative risk estimates alone would suggest.

Our findings extend prior work in young cancer populations. Henson et al. reported elevated cardiac mortality among five-year survivors diagnosed at ages 15 to 39 [1], Wang et al. demonstrated increased cardiovascular mortality in a large American AYA cohort [2], and the TYACSS cohort identified elevated cerebrovascular risk [70]. The most recent comparator by Li et al. synthesized cardiovascular risk across 160 studies of cancer survivors of all ages and identified younger age at diagnosis as a moderator (HR, 2.97 for ages < 20 years; 1.48 for ages 20–49 years) [5]. That synthesis pooled a composite cardiovascular disease outcome but did not develop condition-specific estimates or address cardiovascular mortality present in our analysis.

### Limitations

Heterogeneity was substantial across pooled outcomes, likely reflecting differences in age definitions, cancer types, treatment eras, follow-up duration, comparators, and outcome ascertainment. Although directional consistency was preserved, the magnitude of some estimates warrants cautious interpretation. Certainty of evidence was rated low for most outcomes, primarily reflecting heterogeneity rather than individual-study bias, which was mostly low (Tables S1 and S2).

Comparator groups varied across studies, each carrying different assumptions about baseline risk and confounding that may contribute to heterogeneity. Several large contributing studies derive from the Childhood Cancer Survivor Study (CCSS) cohort, with overlap most concentrated in the cardiovascular mortality pool (14 publications contributing distinct mortality endpoints; Table S5); apparent precision of pooled mortality estimates may therefore be inflated relative to fully independent cohorts.

Treatment-stratified sub-analysis was not feasible: most included studies reported heterogeneous treatment groups within the same cohort, generating pooled estimates reflecting the overall study population rather than specific exposures. We therefore could not determine whether elevated risk is uniform across young patients with cancer or driven primarily by specific cardiotoxic therapies. Cancer type-stratified analysis was similarly not undertaken: although some studies reported cancer type-specific effect estimates, the cancer types represented varied widely across the evidence base, and stratification would have yielded categories too sparse and heterogeneous for meaningful pooling. We therefore could not determine which malignancies contribute disproportionately to the observed excess cardiovascular risk. Effect measures (HR, IRR, RR, SMR) were pooled separately to preserve time-at-risk structure. As pre-specified in the registered protocol, we intended to evaluate risk according to age group, cancer type, treatment era, and follow-up duration; these subgroup analyses were precluded by insufficient or inconsistent reporting of these variables and overlapping category definitions across included studies, limiting our ability to identify the subgroups at highest risk. Egger’s test detected publication bias for cardiovascular mortality, suggesting the pooled SMR may overestimate absolute risk, though directional robustness is supported by consistency with condition-level estimates.

Many included studies predate modern cardioprotective strategies, so absolute risk in contemporary populations may be lower than pooled estimates suggest. The evidence base is predominantly retrospective and registry-based, with limited representation of non-Western and lower-income populations. The search was restricted to English-language studies, which may have introduced language bias by excluding relevant non-English literature. Author contact and hand-searching of reference lists were not performed, which may have resulted in missed eligible studies not captured by the database search strategy. Finally, our analysis quantifies relative risk and does not establish whether earlier or intensified surveillance improves outcomes.

## 5. Conclusions

Pooled observational evidence indicates a substantially elevated risk of cardiovascular mortality among young patients with cancer, with elevated risk across structural, thromboembolic, cerebrovascular, and arrhythmic disease domains compared with age-matched populations. Certainty of evidence was low for most outcomes, reflecting substantial heterogeneity; findings should be interpreted as evidence of direction of risk, with the true magnitude uncertain. Cancer type-stratified analysis was not feasible, so the malignancies and treatment exposures driving this excess risk could not be determined. These findings identify cardiovascular disease as a substantial late effect in this population, though whether earlier or more intensive surveillance improves outcomes remains unproven. Prospective, cancer type- and treatment-era-specific studies are needed, along with broader inclusion of underrepresented populations.

## Figures and Tables

**Table 1:** Summary of pooled meta-analysis results, grouped by cardiovascular domain.

Outcome	Effect Estimate	Number of Studies	Effect (95%CI)	*p*-Value (of Effect)	I^2^
**Conditions**					
Overall Cardiovascular Disease	HR	6	1.7268 [1.2292 to 2.4258] **	0.0016	96.0%
	IRR	5	2.2016 [2.0522 to 2.3618]	<0.0001	44.5%
	RR	5	2.3735 [1.4081 to 4.0009] **	0.0012	95.2%
**Structural**					
Heart failure	HR	9	4.0808 [2.1986 to 7.5744] **	<0.0001	92.5%
	RR	5	9.4103 [2.9217 to 30.3096]	0.0002	97.5%
Cardiomyopathy	HR	6	3.5230 [2.2054 to 5.6275] **	<0.0001	75.9%
	IRR	3	3.3794 [2.6833 to 4.2561]	<0.0001	65.0%
Valvular heart disease	HR	5	2.4127 [1.3054 to 4.4593] **	0.0049	80.1%
Pericardial disease	HR	4	2.4093 [1.2391 to 4.6843]	0.0095	75.4%
**Atherosclerotic/Ischemic**					
Ischemic heart disease	HR	4	1.6588 [0.8162 to 3.3715]	0.1619	92.1%
Myocardial infarction	HR	6	1.4016 [0.6863 to 2.8625]	0.3541	70.5%
**Arrhythmic**					
Cardiac arrhythmia	HR	8	1.3740 [1.1932 to 1.5821]	<0.0001	56.1%
	RR	5	1.4835 [0.9802 to 2.2450] **	0.0621	81.9%
**Cerebrovascular**					
Stroke	HR	5	1.3290 [1.0960 to 1.6117]	0.0038	18.5%
	IRR	3	2.6790 [2.2230 to 3.2286]	<0.0001	57.6%
	RR	3	13.4783 [4.3897 to 41.3845]	<0.0001	80.2%
Cerebrovascular disease	HR	4	2.1267 [0.9193 to 4.9199]	0.0778	91.5%
**Thromboembolic**					
Pulmonary embolism	HR	3	4.6257 [3.0324 to 7.0560]	<0.0001	0.0%
Venous Thrombosis	HR	5	4.0599 [2.5068 to 6.5752]	<0.0001	94.1%
**Other**					
Hypertension	RR	4	1.2906 [1.0987 to 1.5161]	0.0019	40.4%
	IRR	3	1.3414 [1.2484 to 1.4413]	<0.0001	0.0%
**Mortality**					
**Overall**					
Cardiovascular mortality	SMR	18	4.2996 [3.0573 to 6.0468] **	<0.0001	99.1%
Circulatory system disease mortality	SMR	8	3.2888 [2.1176 to 5.1077] **	<0.0001	95.1%
**Cause-specific**					
Cardiac ischemia mortality	SMR	4	3.1520 [1.0493 to 9.4688]	0.0408	98.7%
Cardiomyopathy mortality *	SMR	3	8.0973 [0.9658 to 67.8895]	0.0539	99.1%
Cerebrovascular mortality	SMR	4	2.4392 [1.1683 to 5.0923]	0.0176	96.7%

* Estimate based on three studies with extreme heterogeneity; interpreted descriptively. HR = Hazard Ratio; IRR = Incidence Rate Ratio; SMR = Standardized Mortality Ratio; RR = Risk Ratio. ** Relevant prediction intervals are presented in the Results section.

## Data Availability

All data analyzed in this study are derived from previously published studies. The extracted datasets are available from the corresponding author upon reasonable request.

## Supplementary Materials

The following supporting information can be downloaded at: https://www.mdpi.com/article/10.3390/cancers18213579/s1, Figure S1: Preferred Reporting Items for Systematic Reviews and Meta-Analyses (PRISMA) flowchart of included studies; Figure S2: Forest plot of heart failure; Figure S3: Forest plot of cardiomyopathy; Figure S4: Forest plot of cardiac arrhythmias; Figure S5: Forest plot of ischemic heart disease; Figure S6: Forest plot of myocardial infarction; Figure S7: Forest plot of valvular heart disease; Figure S8: Forest plot of pericardial heart disease; Figure S9: Forest plot of stroke; Figure S10: Forest plot of cerebrovascular disease; Figure S11: Forest plot of pulmonary embolism; Figure S12: Forest plot of thrombosis (venous); Figure S13: Forest plot of hypertension; Table S1: Quality assessment of the included cohort studies using the Newcastle–Ottawa scale; Table S2: GRADE assessment of certainty of evidence; Table S3: Baseline study characteristics for cardiac conditions; Table S4: Baseline study characteristics for cardiac mortality; Table S5: Multi-publication cohort sources contributing to the meta-analysis; Table S6: Reconciliation of unique studies and person-outcome totals contributing to each outcome; Table S7: Results of non-pooled data; Table S8: Sensitivity analysis results; Table S9: Results of meta-regression analysis for cardiovascular mortality.

## Abbreviations

The following abbreviations are used in this manuscript:

AYA	Adolescent and Young Adult
CCSS	Childhood Cancer Survivor Study
CI	Confidence Interval
CVSS	Cardiac and Vascular Late Sequelae in Long-Term Survivors of Childhood Cancer Study
CVD	Cardiovascular Disease
GLMM	Generalized Linear Mixed Model
GRADE	Grading of Recommendations Assessment, Development and Evaluation
HR	Hazard Ratio
IRR	Incidence Rate Ratio
MOOSE	Meta-Analysis Of Observational Studies in Epidemiology
NOS	Newcastle–Ottawa Scale
PRISMA	Preferred Reporting Items for Systematic Reviews and Meta-Analyses
PROSPERO	International Prospective Register of Systematic Reviews
REML	Restricted Maximum Likelihood
RR	Risk Ratio
SE	Standard Error
SHR	Standardized Hospitalization Ratio
SIR	Standardized Incidence Ratio
SMR	Standardized Mortality Ratio
TYACSS	Teenage and Young Adult Cancer Survivor Study
USA	United States of America

## Appendix A

**Table A1:** MOOSE checklist for reporting of meta-analyses of observational studies.

Reporting Criteria	Reported (Yes/No)	Reported on Page No.
**Reporting of Background**		
Problem definition	**Yes**	1–2
Hypothesis statement	**Yes**	1–2
Description of Study Outcome(s)	**Yes**	1–2
Type of exposure or intervention used	**Yes**	1–2
Type of study design used	**Yes**	1–2
Study population	**Yes**	1–2
**Reporting of Search Strategy**		
Qualifications of searchers (e.g., librarians and investigators)	**Yes**	2–3
Search strategy, including time period included in the synthesis and keywords	**Yes**	Appendix B
Effort to include all available studies, including contact with authors	**No**	
Databases and registries searched	**Yes**	2–3
Search software used, name and version, including special features used (e.g., explosion)	**No**	
Use of hand searching (e.g., reference lists of obtained articles)	**No**	
List of citations located and those excluded, including justification	**Yes**	Figure S1; Tables S3 and S4
Method for addressing articles published in languages other than English	**Yes**	3
Method of handling abstracts and unpublished studies	**Yes**	3
Description of any contact with authors	**No**	
**Reporting of Methods**		
Description of relevance or appropriateness of studies assembled for assessing the hypothesis to be tested	**Yes**	3–4
Rationale for the selection and coding of data (e.g., sound clinical principles or convenience)	**Yes**	3–4
Documentation of how data were classified and coded (e.g., multiple raters, blinding, and interrater reliability)	**Yes**	3–4
Assessment of confounding (e.g., comparability of cases and controls in studies where appropriate	**Yes**	3–4; Table S1
Assessment of study quality, including blinding of quality assessors; stratification or regression on possible predictors of study results	**Yes**	3–4; Table S2
Assessment of heterogeneity	**Yes**	3–4; Table S2
Description of statistical methods (e.g., complete description of fixed or random effects models, justification of whether the chosen models account for predictors of study results, dose–response models, or cumulative meta-analysis) in sufficient detail to be replicated	**Yes**	3–4
Provision of appropriate tables and graphics	**Yes**	Table 1; Figure 1 and Figure 2; Tables S1–S8, Figures S1–S13
**Reporting of Results**		
Table giving descriptive information for each study included	**Yes**	Table 1; Tables S3 and S4
Results of sensitivity testing (e.g., subgroup analysis)	**Yes**	9
Indication of statistical uncertainty of findings	**Yes**	9
**Reporting of Discussion**		
Quantitative assessment of bias (e.g., publication bias)	**Yes**	9
Justification for exclusion (e.g., exclusion of non–English-language citations)	**Yes**	3
Assessment of quality of included studies	**Yes**	Table S1
**Reporting of Conclusions**		
Consideration of alternative explanations for observed results	**Yes**	9–10
Generalization of the conclusions (i.e., appropriate for the data presented and within the domain of the literature review)	**Yes**	10
Guidelines for future research	**Yes**	10
Disclosure of funding source	**Yes**	1

## Appendix B. Detailed Search Strategy

*PubMed*

(“Neoplasms”[MeSH] OR cancer*[tiab] OR malignan*[tiab]) AND (“Adolescent”[MeSH] OR “Young Adult”[MeSH] OR adolescent*[tiab] OR “young adult*”[tiab] OR AYA[tiab] OR “early onset”[tiab] OR “early-onset”[tiab] OR “young patient*”[tiab] OR “younger adult*”[tiab]) AND (“Cardiovascular Diseases”[MeSH] OR “Heart Failure”[MeSH] OR “Myocardial Infarction”[MeSH] OR “Stroke”[MeSH] OR “Arrhythmias, Cardiac”[MeSH] OR cardiomyopath*[tiab] OR “heart failure”[tiab] OR “myocardial infarction”[tiab] OR stroke[tiab] OR arrhythmia*[tiab] OR “cardiovascular mortality”[tiab] OR SMR[tiab] OR SIR[tiab] OR “standardized mortality ratio”[tiab]) AND (“cohort studies”[MeSH] OR registries[MeSH] OR cohort[tiab] OR registry[tiab] OR “population-based”[tiab] OR longitudinal[tiab] OR incidence[tiab] OR “hazard ratio”[tiab] OR “risk ratio”[tiab] OR “incidence rate ratio”[tiab])

*Embase*

(‘neoplasm’/exp OR cancer*:ti,ab OR malignan*:ti,ab) AND (‘adolescent’/exp OR ‘young adult’/exp OR adolescent*:ti,ab OR ‘young adult*’:ti,ab OR AYA:ti,ab OR ‘early onset’:ti,ab OR ‘early-onset’:ti,ab OR ‘young patient*’:ti,ab OR ‘younger adult*’:ti,ab) AND (‘cardiovascular disease’/exp OR ‘heart failure’/exp OR ‘myocardial infarction’/exp OR ‘stroke’/exp OR ‘cardiac arrhythmia’/exp OR cardiomyopath*:ti,ab OR ‘heart failure’:ti,ab OR ‘myocardial infarction’:ti,ab OR stroke:ti,ab OR arrhythmia*:ti,ab OR ‘cardiovascular mortality’:ti,ab OR SMR:ti,ab OR SIR:ti,ab OR ‘standardized mortality ratio’:ti,ab) AND (‘cohort analysis’/exp OR ‘cohort study’/exp OR registry:ti,ab OR ‘disease registry’/exp OR ‘population based’:ti,ab OR longitudinal:ti,ab OR incidence:ti,ab OR ‘hazard ratio’:ti,ab OR ‘risk ratio’:ti,ab OR ‘incidence rate ratio’:ti,ab)

*Web of Science*

TS = ((cancer* OR malignan* OR neoplasm*) AND (adolescent* OR “young adult*” OR AYA OR “early onset” OR “early-onset” OR “young patient*” OR “younger adult*”) AND (“cardiovascular disease” OR “heart failure” OR “myocardial infarction” OR stroke OR arrhythmia* OR cardiomyopath* OR “cardiovascular mortality” OR SMR OR SIR OR “standardized mortality ratio”) AND (cohort OR registry OR “population-based” OR longitudinal OR incidence OR “hazard ratio” OR “risk ratio” OR “incidence rate ratio”))

*Scopus*

TITLE-ABS-KEY((cancer* OR malignan* OR neoplasm*) AND (adolescent* OR “young adult*” OR AYA OR “early onset” OR “early-onset” OR “young patient*” OR “younger adult*”) AND (“cardiovascular disease” OR “heart failure” OR “myocardial infarction” OR stroke OR arrhythmia* OR cardiomyopath* OR “cardiovascular mortality” OR SMR OR SIR OR “standardized mortality ratio”) AND (cohort OR registry OR “population-based” OR longitudinal OR incidence OR “hazard ratio” OR “risk ratio” OR “incidence rate ratio”))
