# Supplementary material for: Excess Cardiovascular Burden Among Young Patients with Cancer: A Systematic Review and Meta-Analysis
Source: Cancers (Basel). 2026 Nov 7;18(21):3579. doi: 10.3390/cancers18213579 (PMC13761766; doi:10.3390/cancers18213579)
Supplement: Supplementary file 1 [file cancers-18-03579-s001.zip › cancers-4398707-supplementary.pdf]

Figure S1. Preferred Reporting Items for Systematic Reviews and Meta-Analyses (PRISMA) flowchart of included studies.
Figure S2. Forest plot of heart failure.
Figure S3. Forest plot of cardiomyopathy.
Figure S4. Forest plot of cardiac arrhythmias.
Figure S5. Forest plot of ischemic heart disease.
Figure S6. Forest plot of myocardial infarction.
Figure S7. Forest plot of valvular heart disease.
Figure S8. Forest plot of pericardial heart disease.
Figure S9. Forest plot of stroke.
Figure S10. Forest plot of cerebrovascular disease.
Figure S11. Forest plot of pulmonary embolism.
Figure S12. Forest plot of thrombosis (venous).
Figure S13. Forest plot of hypertension.
Table S1. Quality assessment of the included cohort studies using the Newcastle-Ottawa scale.
Table S2. GRADE assessment of certainty of evidence.
Table S3. Baseline study characteristics for cardiac conditions.
Table S4. Baseline study characteristics for cardiac mortality.
Table S5. Multi-publication cohort sources contributing to the meta-analysis.
Table S6. Reconciliation of unique studies and person-outcome totals contributing to each outcome.
Table S7. Results of non-pooled data.
Table S8. Sensitivity analysis results.
Table S9. Results of meta-regression analysis for cardiovascular mortality.

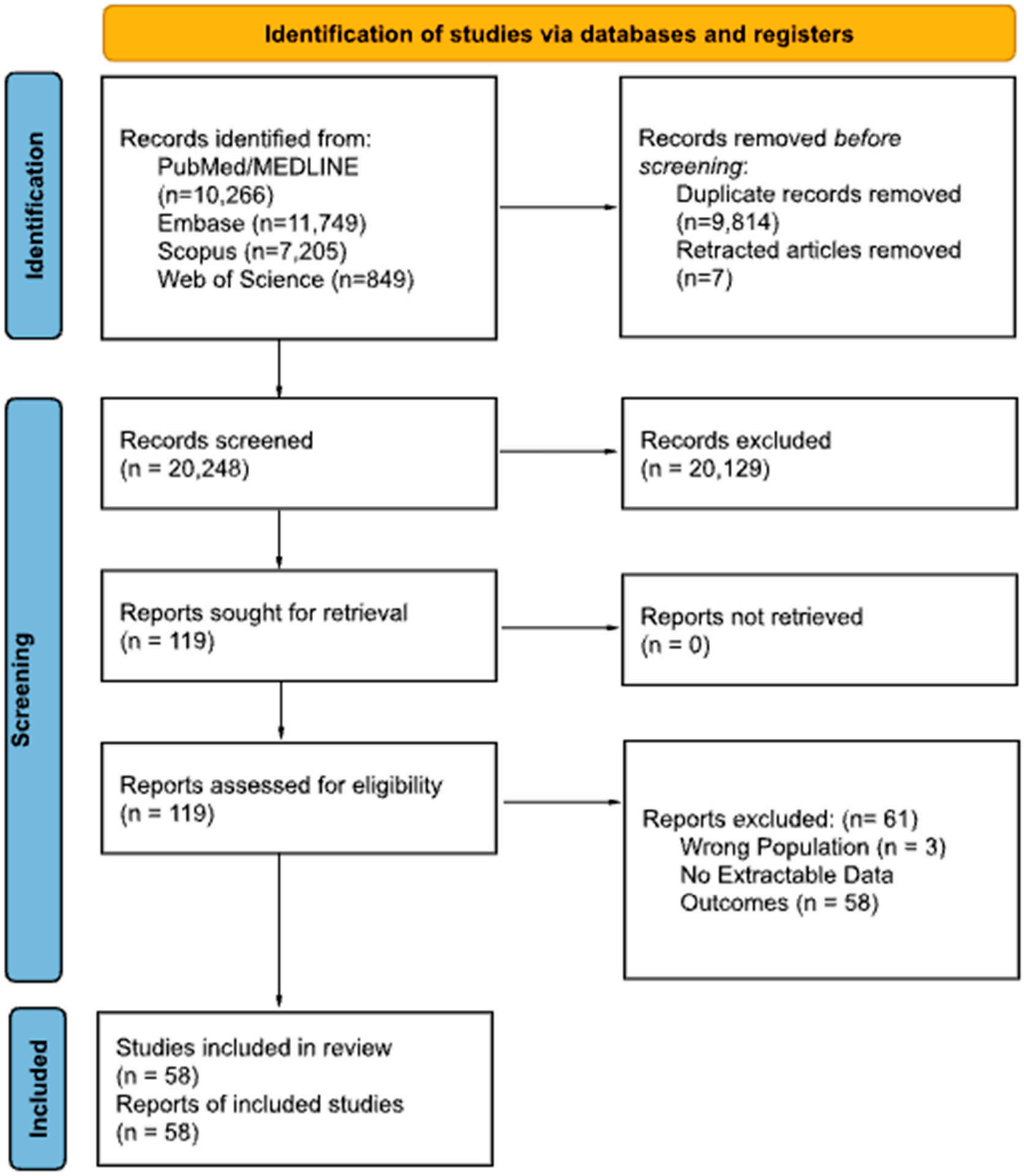

Figure S1. Preferred Reporting Items for Systematic Reviews and Meta-Analyses (PRISMA) flowchart of included studies.

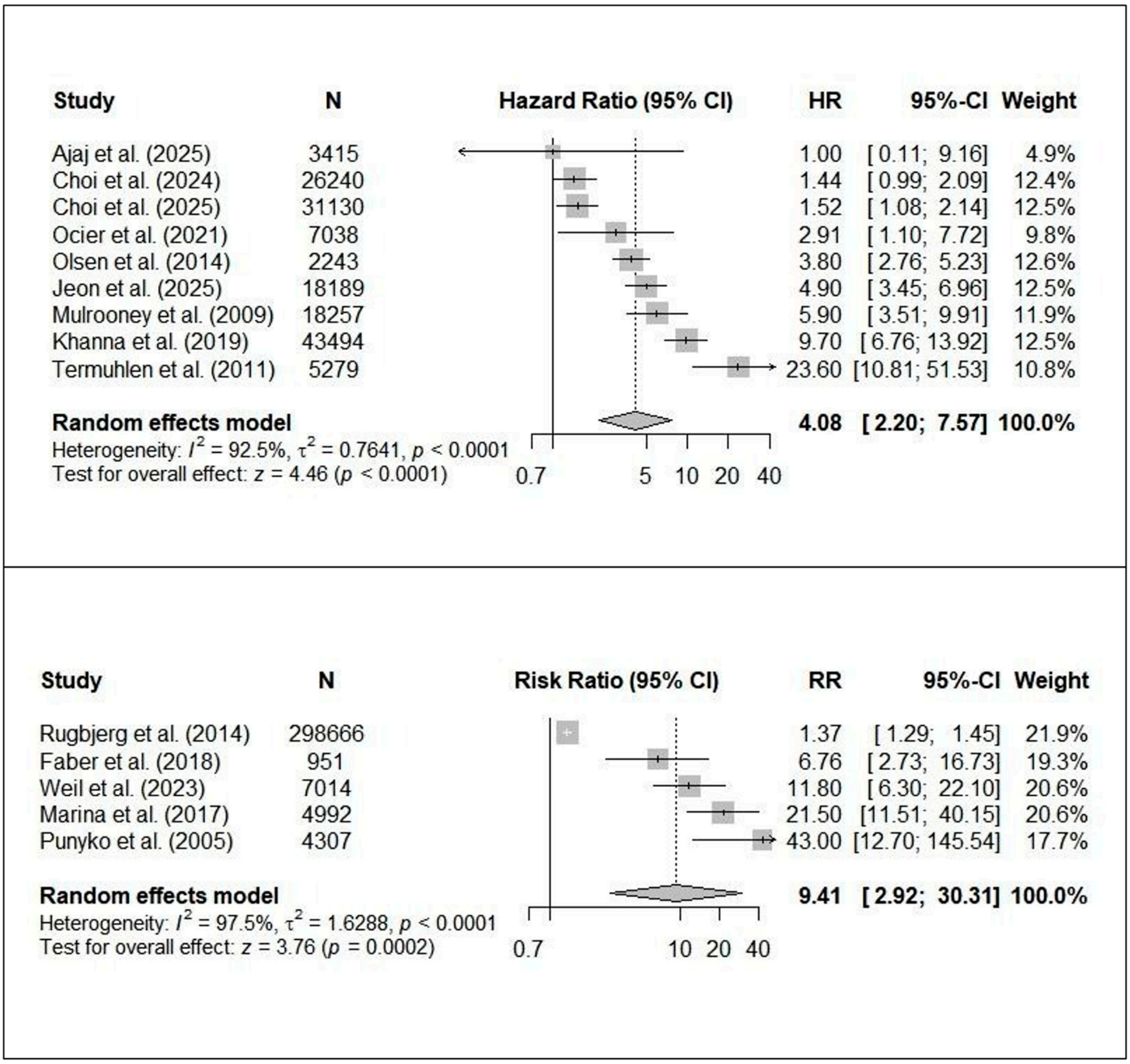

Figure S2. Forest plot of heart failure [17,22–24,28–37].

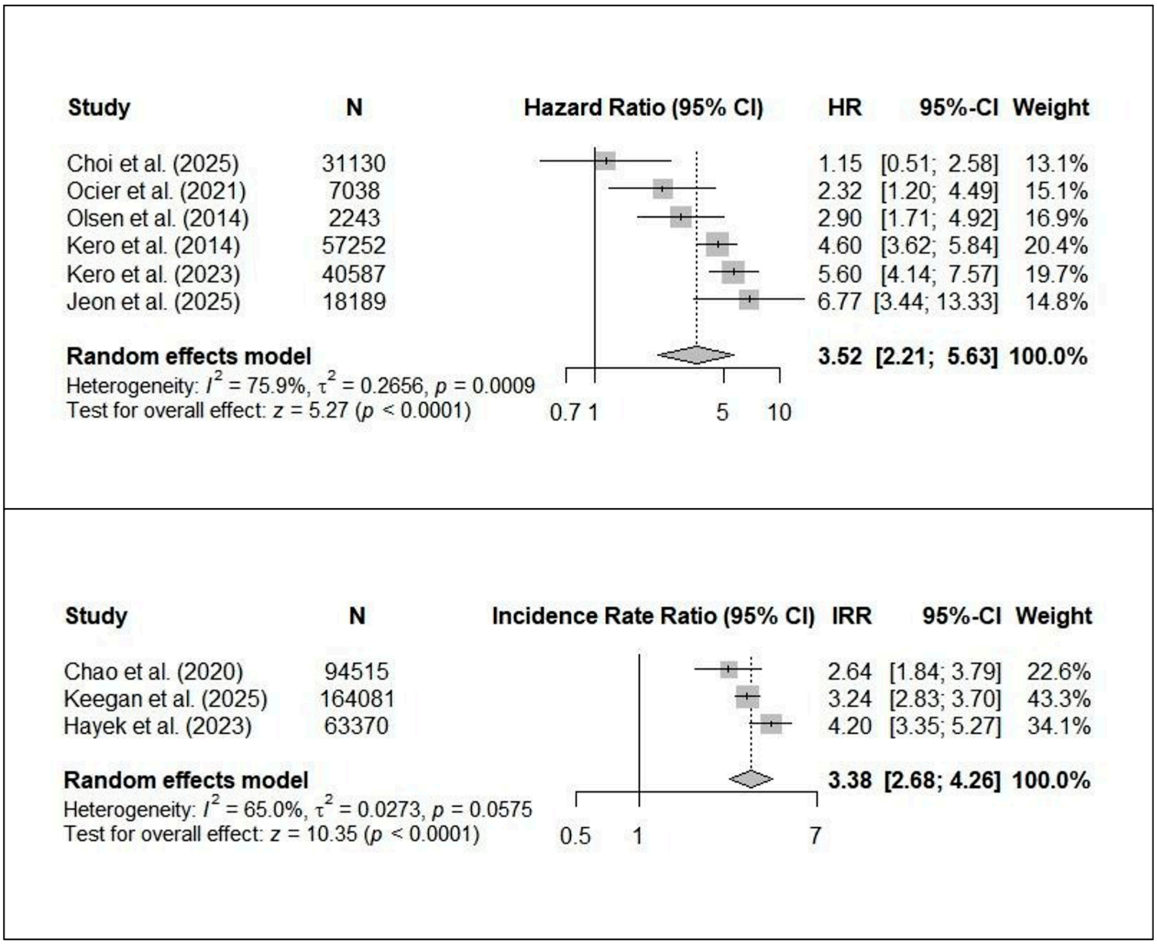

Figure S3. Forest plot of cardiomyopathy [19,23,26,27,30,31,33,38,39].

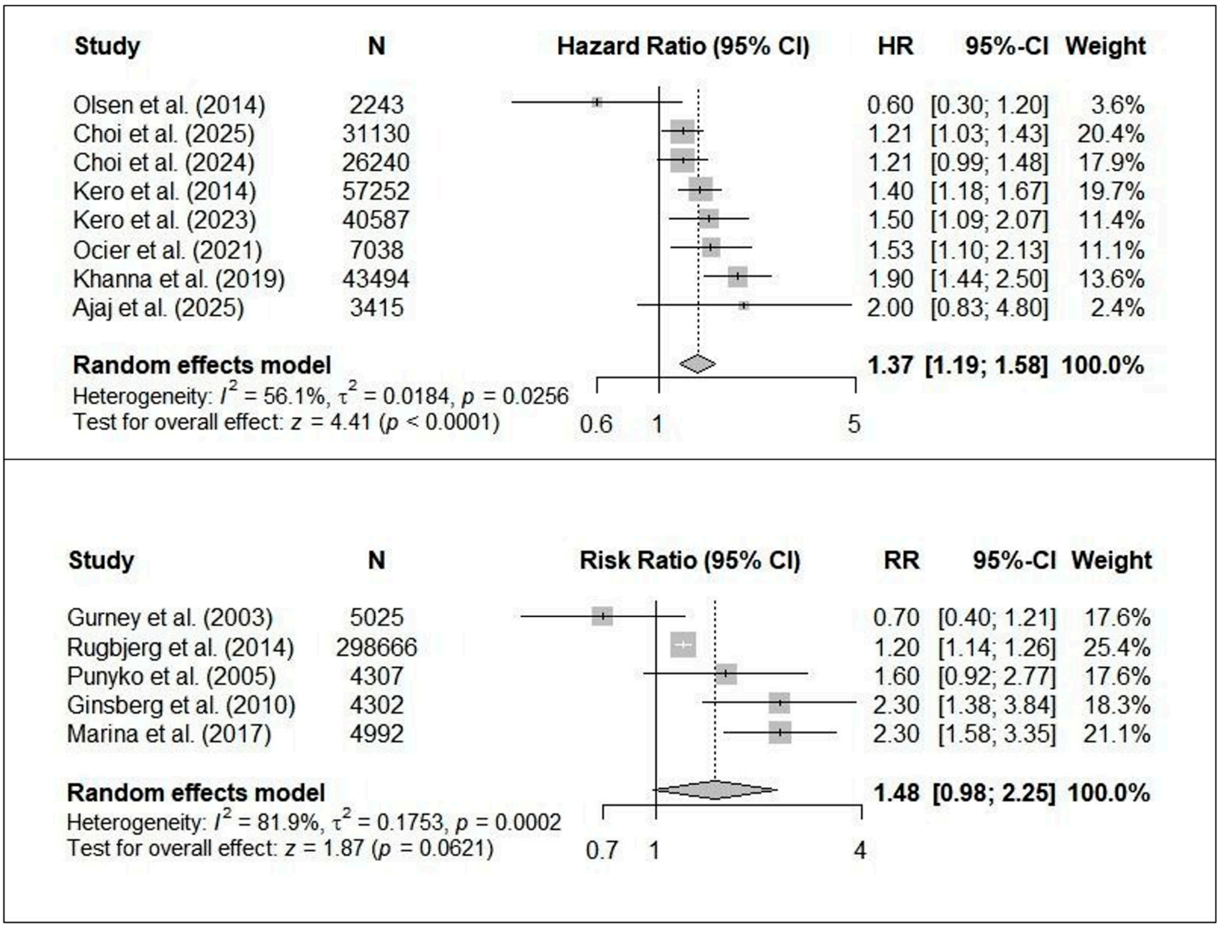

Figure S4. Forest plot of cardiac arrhythmias [17,22,23,25,28-31,32,35,38–40].

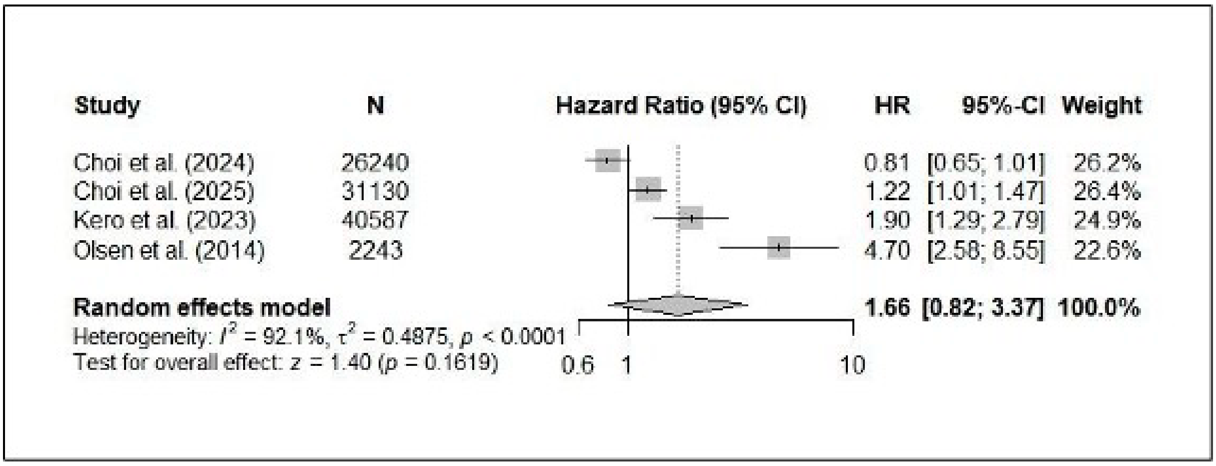

Figure S5. Forest plot of ischemic heart disease [22,23,31,39].

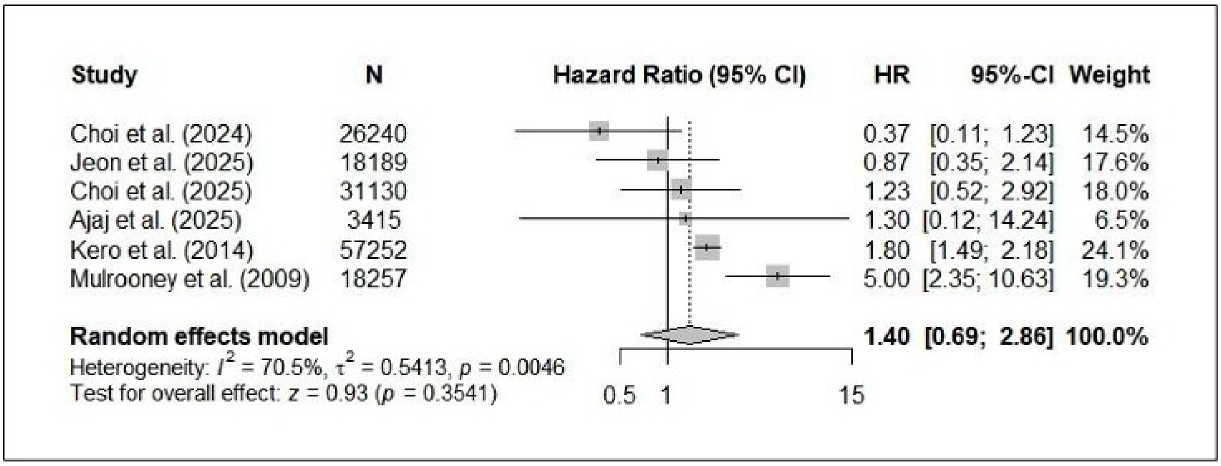

Figure S6. Forest plot of myocardial infarction [17,22,23,33,34,38].

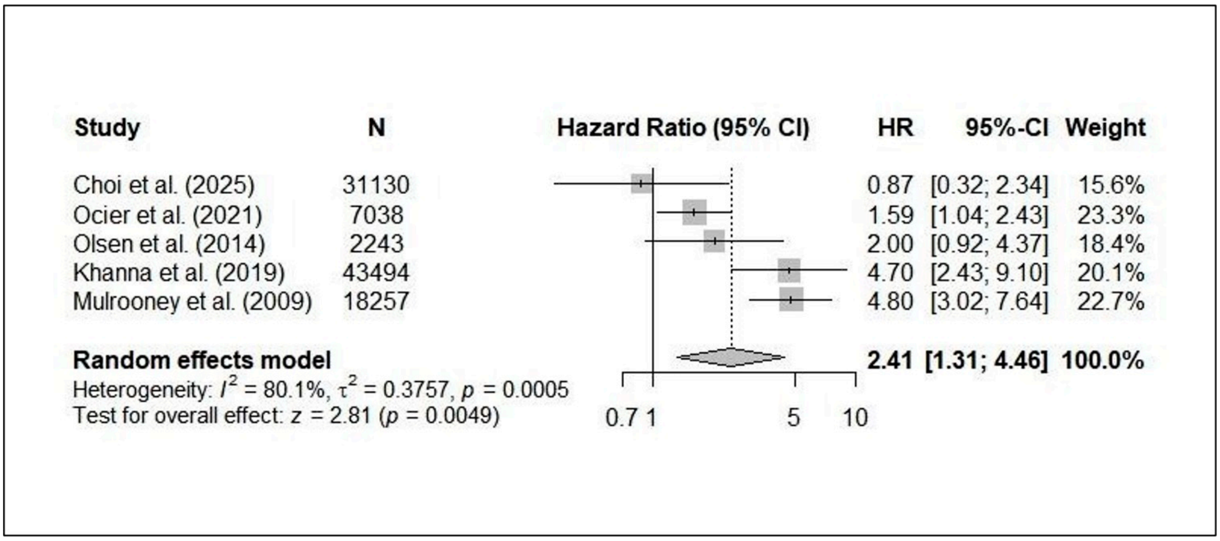

Figure S7. Forest plot of valvular heart disease [23,28,30,31,34].

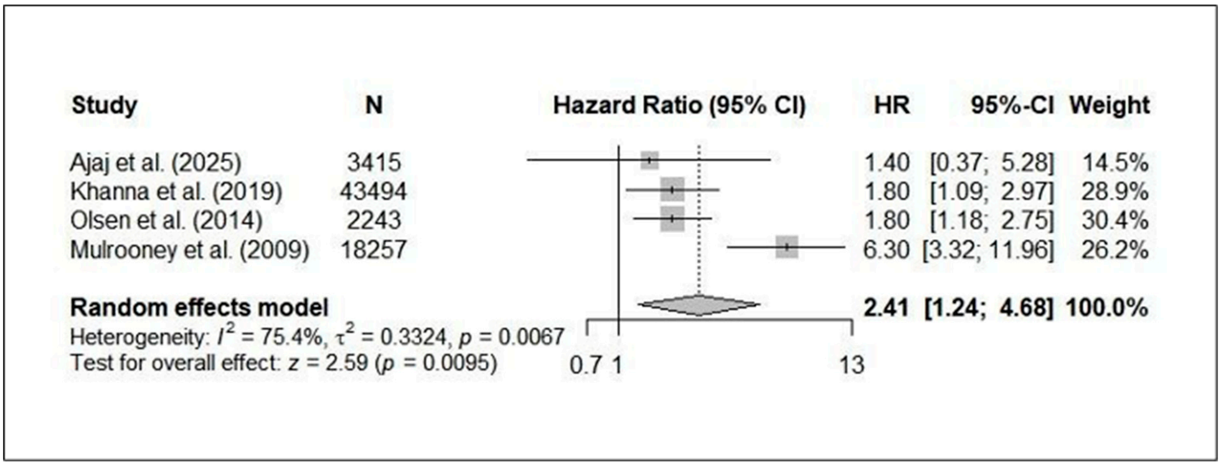

Figure S8. Forest plot of pericardial heart disease [17,28,30,34].

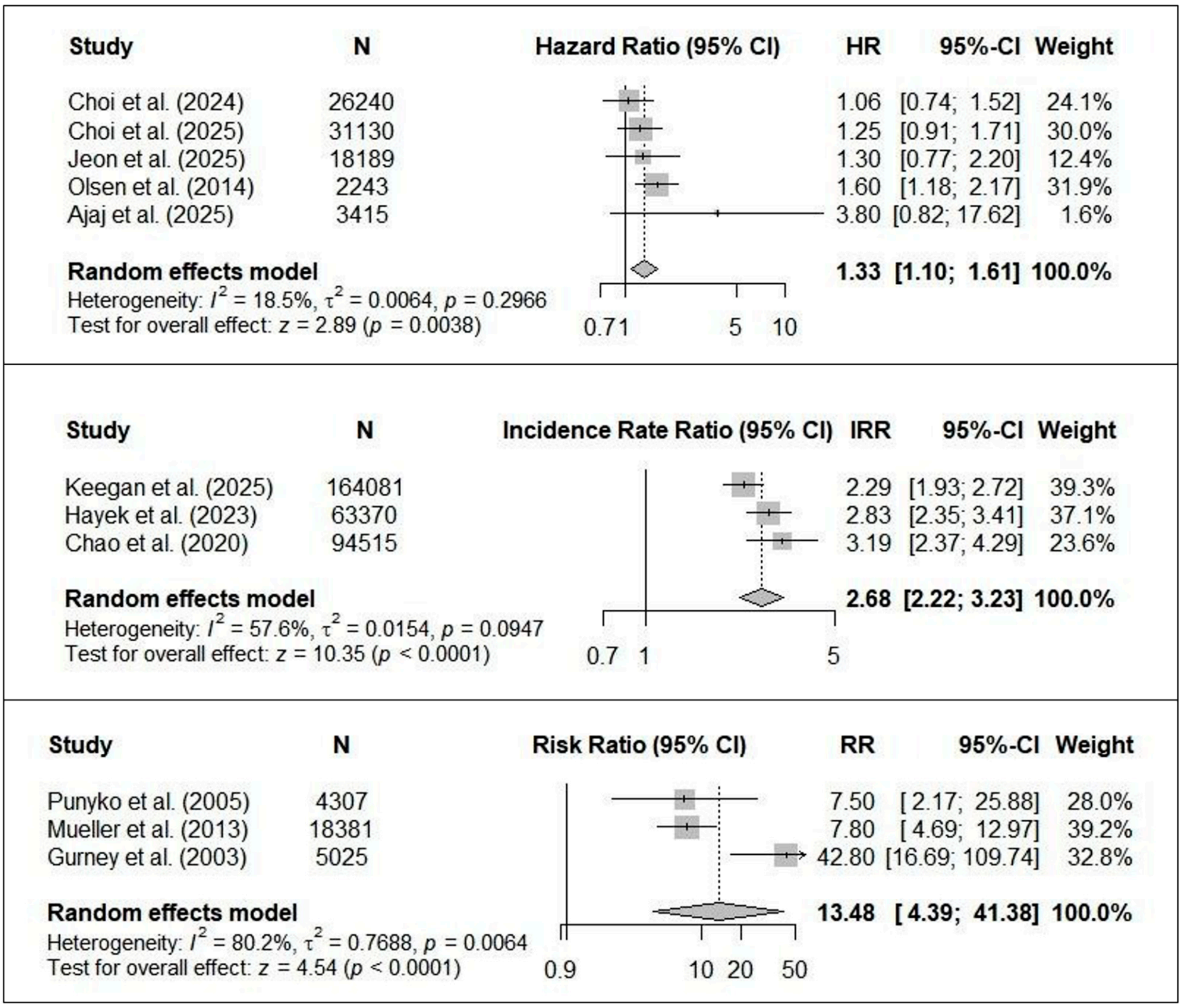

Figure S9. Forest plot of stroke [17,19,22,23,26,27,31,33,35,40,41].

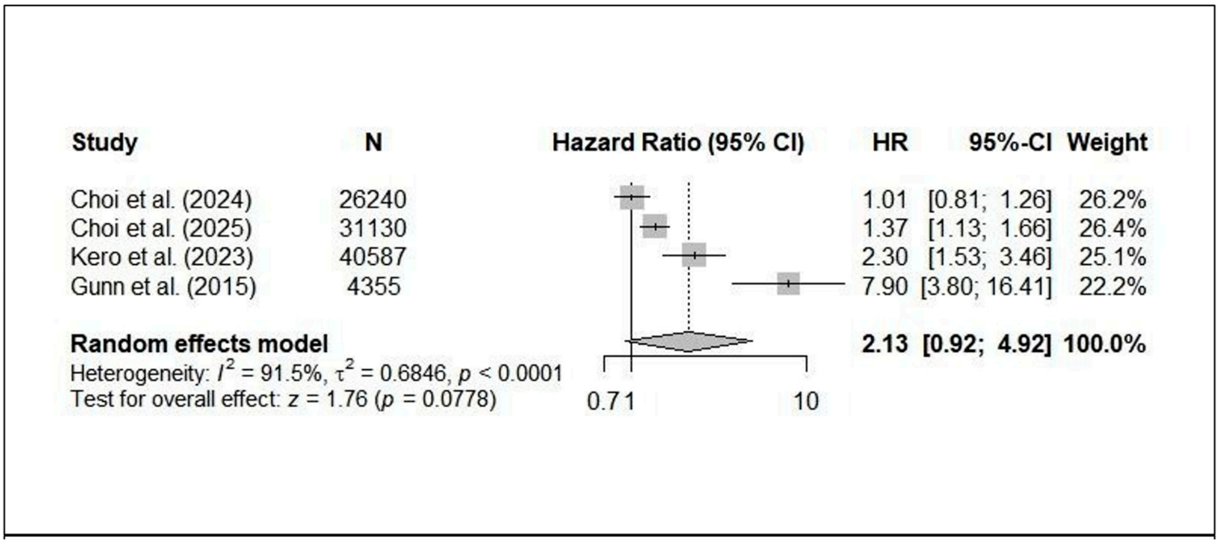

Figure S10. Forest plot of cerebrovascular disease [22,23,39,42].

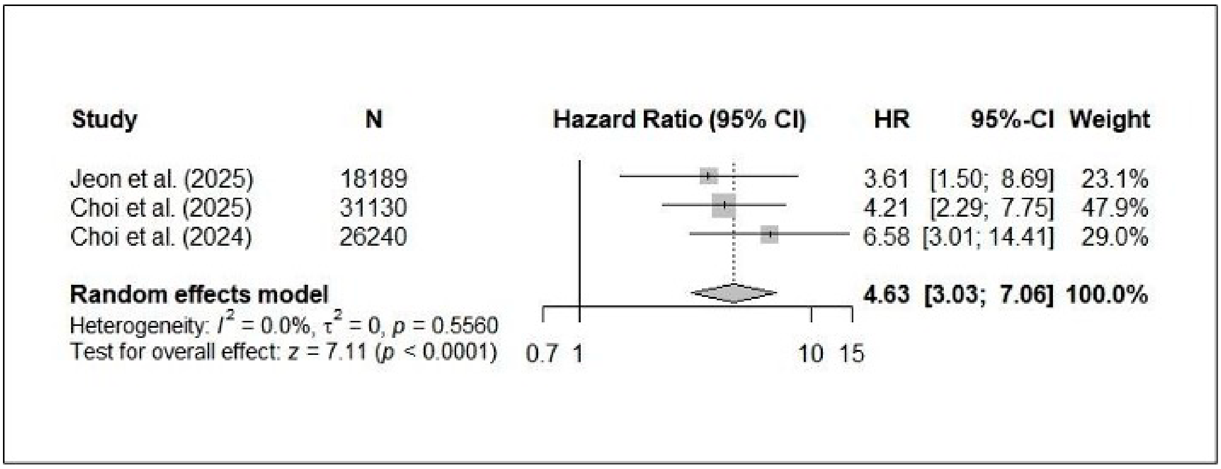

Figure S11. Forest plot of pulmonary embolism [22,23,33].

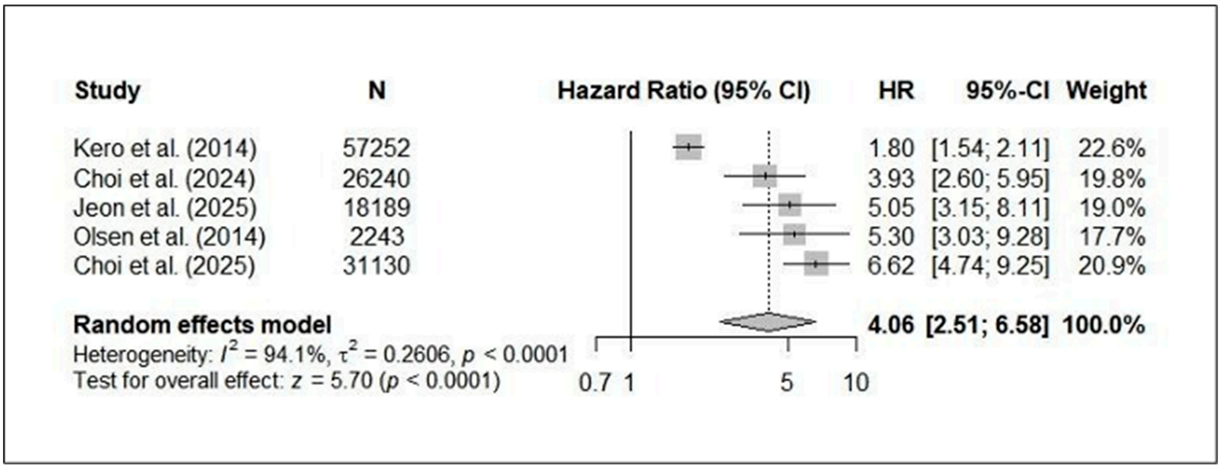

Figure S12. Forest plot of Thrombosis (venous) [22,23,31,33,38].

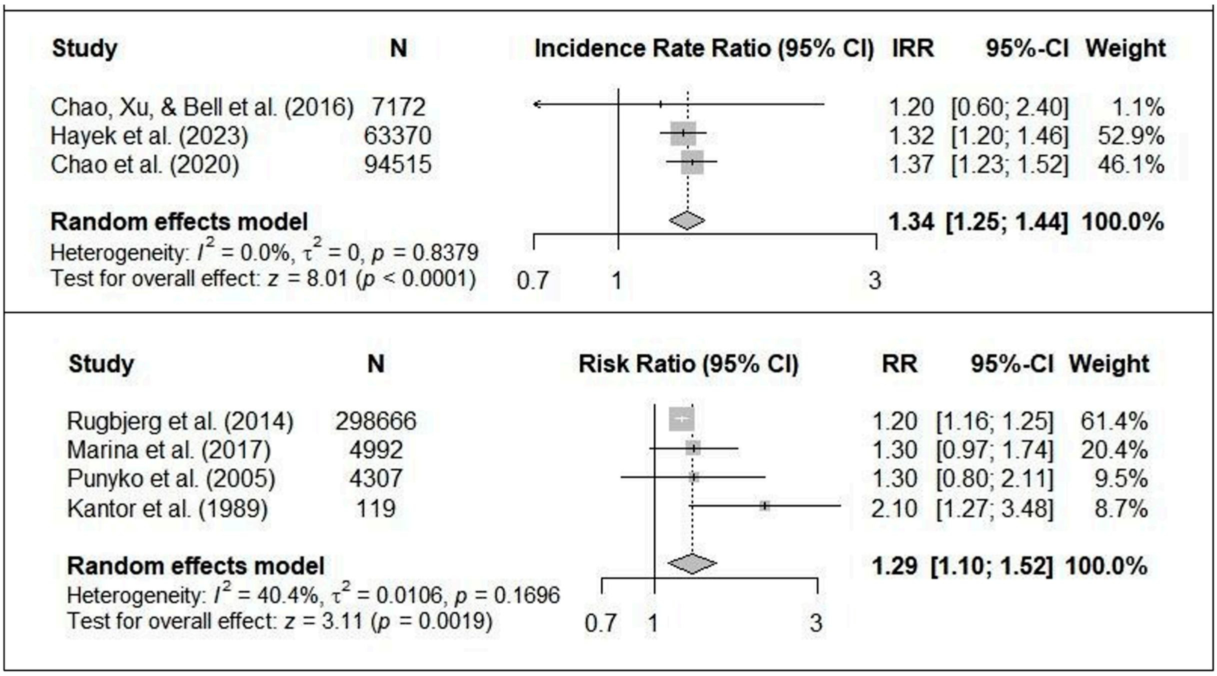

Figure S13. Forest plot of hypertension [19,20,26,29,32,35,43].

Table S1. Quality assessment of the included cohort studies using the Newcastle-Ottawa scale [1,2,4,11–64,70].

First Author, Year	Selection				Comparability	Outcome			Total Score	Risk of Bias
	Representativeness of Exposed Cohort	Selection of Non-Exposed Cohort	Ascertainment of Exposure	Outcome Not Present at Start		Assessment of Outcome	Adequacy of Follow-up Length	Adequacy of Follow-up Completeness		
Ajaj et al. (2025) [17]	⋆	⋆	⋆	⋆	⋆⋆	⋆	-	⋆	8/9	Low
Anderson et al. (2019) [44]	⋆	⋆	⋆	⋆	⋆	⋆	⋆	⋆	8/9	Low
Armstrong et al. (2009) [45]	⋆	⋆	⋆	⋆	⋆	-	⋆	⋆	7/9	Low

Bagnasco et al (2019) [46]	*	*	*	*	*	*	*	*	8/9	Low
Becktell et al. (2024) [18]	*	*	*	*	*	-	*	*	7/9	Low
Bhandari et al. (2025) [47]	*	*	*	*	**	-	*	*	8/9	Low
Bhuller et al. (2016) [11]	*	*	*	-	*	-	*	*	6/9	Moderate
Bottinor et al. (2024) [4]	*	*	*	*	**	-	*	*	8/9	Low
Brewster et al. (2013) [58]	*	*	*	*	*	*	*	*	8/9	Low
Bright et al. (2017) [70]	*	*	*	*	*	*	*	*	8/9	Low
Castellino et al. (2011) [48]	*	*	*	*	*	-	*	*	7/9	Low
Chao et al. (2020) [19]	-	*	*	*	**	*	*	*	8/9	Low
Chao, Xu, & Bell et al. (2016) [20]	-	*	*	*	*	*	*	*	7/9	Low
Chao, Xu, & Bhatia et al. (2016) [21]	-	*	*	*	**	*	*	-	7/9	Low
Choi et al. (2024) [22]	*	*	*	*	**	*	*	*	9/9	Low
Choi et al. (2025) [23]	*	*	*	*	**	*	*	*	9/9	Low
Faber et al. (2018) [24]	*	*	*	-	**	*	*	*	8/9	Low
Fidler et al. (2016) [59]	*	*	*	*	*	*	*	*	8/9	Low
Fidler et al. (2017) [49]	*	*	*	*	*	*	*	*	8/9	Low
Fosså et al. (2004) [60]	*	*	*	*	*	-	*	-	6/9	Moderate
Ginsberg et al. (2010) [25]	*	*	*	*	**	-	*	*	8/9	Low
Gunn et al. (2015) [42]	*	*	*	*	*	*	*	*	8/9	Low
Gurney et al. (2003) [40]	*	*	*	*	*	-	*	*	7/9	Low
Hancock et al. (1993) [12]	-	-	*	*	*	*	*	*	6/9	Moderate

Hayek et al. (2023) [26]	*	*	*	*	**	*	*	*	9/9	Low
Henson et al. (2016) [1]	*	*	*	*	*	*	*	*	8/9	Low
Jeon et al. (2025) [33]	*	*	*	*	*	*	-	*	7/9	Low
Kantor et al. (1989) [43]	-	-	*	*	*	-	*	*	4/9	High
Keegan et al. (2025) [27]	-	*	*	*	**	*	*	*	8/9	Low
Kero et al. (2014) [38]	*	*	*	*	*	*	*	*	8/9	Low
Kero et al. (2015) [50]	*	*	*	*	*	*	*	*	8/9	Low
Kero et al. (2023) [39]	*	*	*	*	*	*	*	*	8/9	Low
Khanna et al. (2019) [28]	*	*	*	*	**	*	*	*	9/9	Low
Laverdière et al. (2009) [51]	*	*	*	*	*	-	*	*	7/9	Low
MacArthur et al. (2007) [61]	*	*	*	*	*	*	*	*	8/9	Low
Marina et al. (2017) [29]	*	*	*	*	**	-	-	-	6/9	Moderate
McKenzie et al. (2025) [62]	*	*	*	*	**	*	*	*	9/9	Low
Mertens et al. (2001) [53]	*	*	*	*	*	-	*	*	7/9	Low
Mertens et al. (2008) [52]	*	*	*	*	*	-	*	*	7/9	Low
Merzenich et al. (2022) [54]	*	*	*	*	*	-	*	-	7/9	Low
Möller et al. (2001) [55]	*	*	*	*	*	*	*	*	8/9	Low
Mueller et al. (2013) [41]	*	*	*	*	**	*	*	*	9/9	Low
Mulrooney et al. (2009) [34]	*	*	*	*	**	-	*	*	8/9	Low
Ocier et al. (2021) [30]	*	*	*	*	**	*	*	*	9/9	Low
Olsen et al. (2014) [31]	*	*	*	*	*	-	*	*	7/9	Low

Pedersen et al. (2021) [13]	*	*	*	*	**	*	*	*	9/9	Low
Prasad et al. (2012) [63]	*	*	*	*	*	*	*	*	8/9	Low
Punyko et al. (2005) [35]	*	*	*	*	*	-	*	*	7/9	Low
Reulen et al. (2010) [56]	*	*	*	*	**	*	*	*	9/9	Low
Reulen et al. (2021) [14]	*	-	*	*	*	*	*	*	7/9	Low
Rugbjerg et al. (2014) [32]	*	*	*	*	*	*	*	*	8/9	Low
Schindler et al. (2016) [57]	*	*	*	*	*	*	*	*	8/9	Low
Termuhlen et al. (2011) [36]	*	*	*	*	**	-	*	*	8/9	Low
Trama et al. (2022) [15]	*	*	*	*	*	*	*	*	8/9	Low
Van Den Belt-Dusebout et al. (2006) [16]	*	*	*	*	**	*	*	*	9/9	Low
Wang et al. (2021) [2]	*	*	*	*	*	*	*	*	8/9	Low
Weil et al. (2023) [37]	*	*	*	*	**	-	*	*	8/9	Low
Youlden et al. (2021) [64]	*	*	*	*	*	*	*	*	8/9	Low

Table S2. GRADE assessment of certainty of evidence.

Outcome	No. of Studies	No. of Participants	Risk of Bias[a]	Inconsistency[b]	Indirectness[c]	Imprecision[d]	Publication Bias[e]	Certainty of Evidence	Pooled Effect Estimate (95% CI)
**Cardiovascular Conditions**									
**Overall CVD**	16	821,207	Serious (-1) Included self-	Very serious (-2) HR I^2 96%; IRR I^2	Not serious	Not serious	Publication bias cannot be excluded;	**Very Low**	*HR 1.73 (1.23–2.43); IRR 2.20 (2.05–2.36); RR 2.37 (1.41–4.00)*

			reported CCSS outcomes	44.5%; RR I^2 95.2%			not formally assessed due to <10 studies		
**Heart Failure**	15	473,554	Serious (-1) Included self-reported CCSS outcomes	Very serious (-2) HR I^2 92.5%; RR I^2 97.5%	Not serious	Not serious	Publication bias cannot be excluded; not formally assessed due to <10 studies	**Very Low**	*HR 4.08 (2.20–7.57); RR 9.41 (2.92–30.31); IRR 3.38 (2.68–4.26)*
**Cardiomyopathy**	10	777,071	Not serious All registry or claims-based	Serious (-1) HR I^2 75.9%; IRR I^2 65%	Not serious	Not serious	Publication bias cannot be excluded; not formally assessed due to <10 studies	**Low**	*HR 3.52 (2.21–5.63); IRR 3.38 (2.68–4.26)*
**Cardiac Arrhythmia**	14	692,772	Serious (-1) Included self-reported CCSS outcomes	Serious (-1) HR I^2 56%; RR I^2 82%	Not serious	Serious (-1)	Publication bias cannot be excluded; not formally assessed due to <10 studies	**Very Low**	*HR 1.37 (1.19–1.58); RR 1.48 (0.98–2.25)*
**Ischemic Heart Disease**	6	462,236	Not serious All registry or claims-based	Very serious (-2) HR I^2 92.1%	Not serious	Serious (-1)	Publication bias cannot be excluded; not formally assessed due to <10 studies	**Very Low**	*HR 1.66 (0.82–3.37)*
**Myocardial Infarction**	9	166,121	Serious (-1) Included self-reported CCSS outcomes	Serious (-1) HR I^2 70.5%	Not serious	Serious (-1)	Publication bias cannot be excluded; not formally assessed due to <10 studies	**Very Low**	*HR 1.40 (0.69–2.86)*

**Valvular Heart Disease**	7	271,235	Serious (-1) Included self-reported CCSS outcomes	Very serious (-2) HR I² 80.1%	Not serious	Not serious	Publication bias cannot be excluded; not formally assessed due to <10 studies	**Very Low**	*HR 2.41 (1.31–4.46)*
**Pericardial Disease**	5	231,490	Serious (-1) Included self-reported CCSS outcomes	Very serious (-2) HR I² 75.4%	Not serious	Not serious	Publication bias cannot be excluded; not formally assessed due to <10 studies	**Very Low**	*HR 2.41 (1.24–4.68)*
**Stroke**	12	609,822	Serious (-1) Included self-reported CCSS outcomes	Very serious (-2) HR I² 18.5%; IRR I² 57.6%; RR I² 80.2%	Not serious	Not serious	Publication bias cannot be excluded; not formally assessed due to <10 studies	**Very Low**	*HR 1.33 (1.10–1.61); IRR 2.68 (2.22–3.23); RR 13.48 (4.39–41.38)*
**Cerebrovascular disease**	9	602,872	Not serious All registry or claims-based	Very serious (-2) HR I² 91.5%	Not serious	Serious (-1)	Publication bias cannot be excluded; not formally assessed due to <10 studies	**Very Low**	*HR 2.13 (0.92–4.92)*
**Pulmonary Embolism**	3	75,559	Not serious All registry/claims-based	Not serious HR I² 0%	Not serious	Not serious	Publication bias cannot be excluded; not formally assessed due to <10 studies	**Low**	*HR 4.63 (3.03–7.06)*
**Venous Thrombosis**	6	140,079	Not serious All registry or	Very serious (-2) HR I² 94.1%	Not serious	Not serious	Publication bias cannot be excluded;	**Low**	*HR 4.06 (2.51–6.58)*

			claims-based				not formally assessed due to <10 studies		
**Hypertension**	9	481,835	Not serious All registry or claims-based	Not serious RR I² 40.4%; IRR I² 0%	Not serious	Not serious	Publication bias cannot be excluded; not formally assessed due to <10 studies	**Moderate**	*RR 1.29 (1.10–1.52); IRR 1.34 (1.25–1.44)*
**Coronary Artery Disease**	5	307,844	Not serious All registry or claims-based	Serious (–1) Effect measure formats heterogeneous; pooling preclude[f]	Not serious	Serious (–1)	Publication bias cannot be excluded; not formally assessed due to <10 studies	**Very Low**	*Not pooled. See Table S7.*
**Circulatory System Disease**	4	16,318	Not serious All registry or claims-based	Serious (–1) Effect measure formats heterogeneous; pooling preclude[f]	Not serious	Serious (–1)	Publication bias cannot be excluded; not formally assessed due to <10 studies	**Very Low**	*Not pooled. See Table S7.*
**Angina/Chest Pain**	4	18,709	Serious (-1) Included self-reported CCSS outcomes	Serious (–1) Effect measure formats heterogeneous; pooling preclude[f]	Not serious	Serious (–1)	Publication bias cannot be excluded; not formally assessed due to <10 studies	**Very Low**	*Not pooled. See Table S7.*
**Cardiovascular Mortality**									

**Overall Cardiovascular Mortality**	18	830,262	Not serious All registry/death index linkage	Very serious (-2) SMR I^2 99.1%	Not serious	Not serious	Suspected (-1) Egger's test significant	**Very Low**	*SMR 4.30 (3.06–6.05)*
**Circulatory Disease Mortality**	8	96,978	Not serious All registry/death index linkage	Very serious (-2) SMR I^2 95.1%	Not serious	Not serious	Publication bias cannot be excluded; not formally assessed due to <10 studies	**Very Low**	*SMR 3.29 (2.12–5.11)*
**Cardiac Ischemia Mortality**	4	298,729	Not serious All registry/death index linkage	Very serious (-2) SMR I^2 98.7%	Not serious	Serious (-1)	Publication bias cannot be excluded; not formally assessed due to <10 studies	**Very Low**	*SMR 3.15 (1.05–9.47)*
**Cardiomyopathy Mortality**	3	238,068	Not serious All registry/death index linkage	Very serious (-2) SMR I^2 99.1%	Not serious	Very serious (-2)	Publication bias cannot be excluded; not formally assessed due to <10 studies	**Very Low**	*SMR 8.10 (0.97–67.89)*
**Cerebrovascular Mortality**	4	417,090	Not serious All registry/death index linkage	Very serious (-2) SMR I^2 96.7%	Not serious	Serious (-1)	Publication bias cannot be excluded; not formally assessed due to <10 studies	**Very Low**	*SMR 2.44 (1.17–5.09)*

Abbreviations: CI, confidence interval; GRADE, Grading of Recommendations Assessment, Development and Evaluation; HR, hazard ratio; IRR, incidence rate ratio; No., number; RR, risk ratio; SMR, standardized mortality ratio.
Per GRADE, observational studies begin at Low certainty and cannot fall below Very Low.

a. Risk of bias: we downgrade by one level when included studies relied on self-reported outcome ascertainment.
b. Inconsistency: we downgrade by one level if $I^2 > 50\%$ and by two levels if $I^2 > 75\%$.
c. Indirectness: we downgrade by one level if there are differences in the PICO of the original studies included between different associations.
d. Imprecision: we downgrade by one level if the 95% CI crosses the null or the lower bound approaches unity.
e. Publication bias: we downgrade by one level if Egger's test $P < 0.05$; this test was performed only for outcomes with ≥10 contributing studies.
f. For outcomes where heterogeneous effect measure formats precluded meta-analytic pooling, certainty was assessed based on the body of individual study estimates summarized in eTable 7. Inconsistency was downgraded by one level given the inability to quantitatively assess between-study variation (I^2 could not be computed).

Table S3. Baseline study characteristics for conditions [11,13–43,70].

Study	Country	Outcome(s)	Comparator Type	Male %	Age Range	Cancer Type (specific site if reported)	Time Since Diagnosis	Follow-up duration (median/ mean; range; person-years if available)	Cancer Treatment Exposure
Ajaj et al. (2025) [17]	Canada	Cardiac arrhythmia; CVD Overall; HF; CAD; HTN; MI; Pericardial Disease; Stroke	Matched general population	NR	NR	Testicular germ cell tumor (TGCT)	≥5 years	Mean: 15.0 (SD 7.0) years	Chemotherapy (cisplatin-based), surgery, radiation, HSCT
Becktell et al. (2024) [18]	USA and Canada	CVD (overall)	Sibling without cancer	51.5	<21	Osteosarcoma	≥5 years	Mean: 24.4 years (range 5.0-45.2)	Chemotherapy regimens (MA, MABCD, MABCDP, MAP, other); anthracyclines, cisplatin, alkylating agents; surgical

									approach (amputation vs limb salvage)
Bhuller et al. (2016) [11]	Canada	Circulatory system disease	General population	50	15-24	Hodgkin lymphoma	≥ 5 years	Median: 19.6 years	Radiation, chemotherapy, surgery or combination; specifics not reported
Bright et al.(2017) [70]	England	Cerebrovascular disease; Stroke (hemorrhagic); Stroke (ischemic)	General population	47.5	15-39	Multiple Malignancies	≥5 years	Median: 11.3 years (range 0-15.8)	Not specified
Chao et al. (2020) [19]	USA	Cardiomyopathy; CVD Overall; CAD; HTN; Stroke;	Matched general population	65.3	15–39	Multiple Malignancies	≥ 2 years	Mean: 6.1 (SD 3.5) years	Radiation therapy: abdominal radiation, chest radiation, head/brain/neck radiation, head/brain radiation, pelvis radiation<br><br>Chemotherapy: alkylating agents, platinum agents, antimetabolite agents (including methotrexate), anthracycline agents, antitumor antibiotics, monoclonal antibodies, corticosteroids
Chao, Xu, & Bell et al. (2016) [20]	USA	CVD (overall); Cerebrovascular disease; HTN	Matched general population	53.2	<18	Multiple Malignancies	≥5 years	Mean: 6.2 years (4.1)	Not specified
Chao, Xu, & Bhatia	USA	CVD (overall)	Matched general populatio	34.9	15-39	Multiple malignancies	≥2 years	Mean: 4.4 (SD 3.4) years	Not specified

et al. (2016) [21]			n						
Choi et al. (2024) [22]	South Korea	Cardiac arrhythmia; CVD (overall); Cerebrovascular disease; HF; Ischemic heart disease; MI; PE; Stroke; Thrombosis (Deep Vein)	Matched general population	44.2	15-39	Gastric cancer	Not specified	Median: 6.5 years	Surgery; surgery plus chemotherapy; surgery plus radiotherapy; endoscopic resection
Choi et al. (2025) [23]	South Korea	Cardiac arrhythmia; CVD (overall); Cerebrovascular disease; HF; Ischemic heart disease; MI; PE; Stroke; Thrombosis (Deep Vein); VHD; Cardiomyopathy	Matched general population	0	15-39	Cervical cancer	Not specified	median: 8 years	Conization; surgery; chemotherapy; radiotherapy; concurrent chemoradiotherapy; platinum based chemotherapy
Faber et al. (2018) [24]	Germany	CVD (overall); HF	General population	55.3	<15	Multiple malignancies	≥5 years	Mean: 28.4 years (range 23-36)	Chemotherapy and radiation
Ginsberg et al. (2010) [25]	USA and Canada	CVD (overall)	Sibling without cancer	52.5	<21	Ewing Sarcoma	≥5 years	Mean: 23.0 years (range 16-33)	Anthracyclines; alkylating agents; ifosfamide; etoposide; radiation therapy; surgery and/or radiation used for local therapy

Gunn et al. (2015) [42]	Finland	Cerebrovascular disease; Circulatory system disease	Sibling without cancer	54.1	<16	Brain Malignancies	≥ 5 years	NR	Not specified
Gurney et al. (2003) [40]	USA and Canada	Angina-like symptoms; Cardiac arrhythmia; Stroke; Thrombosis (Deep vein)	Sibling without cancer	54.3	<21	CNS tumors	≥5 years	NR	Surgery only; surgery + radiation; surgery + radiation + chemotherapy; radiation dose (brain, spine, thyroid); chemotherapy classes including alkylating agents, platinum agents, and others
Hayek et al. (2023) [26]	Israel	Cardiomyopathy; CVD Overall; CAD; Ischemic heart disease; HTN; Stroke; MI; PE; Thrombosis (Deep vein)	Matched general population	38.3	15-39	Multiple malignancies	≥ 2 years	Mean: 7.6 years	Not specified
Jeon et al. (2025) [33]	South Korea	Cardiomyopathy, Heart Failure, Myocardial Infarction, Stroke, Pulmonary Embolism, Venous Thrombosis	Matched general population	43	15-39	Non-Hodgkin lymphoma	Not specified	Median: 6.5 years	Chemotherapy; radiotherapy; hematopoietic stem cell transplantation
Kantor et al. (1989) [43]	USA	HTN	General population	43	<18	Wilms tumor and RCC	Not specified	Median: 25 years (range 14-53)	Unilateral nephrectomy (all); abdominal irradiation; chemotherapy including dactinomycin

Keegan et al. (2025) [27]	USA	Cardiac arrhythmia; Cardiomyopathy; HF; CAD; Pericardial disease; Stroke; VHD	Matched general population	35	15-39	Multiple malignancies	≥ 2 years	Median: 5.9 years (range 2-17.5)	Chemotherapy; radiotherapy; hematopoietic stem cell transplantation; surgery
Kero et al. (2014) [38]	Finland	Cardiac arrhythmia; Cardiomyopathy; MI; Thrombosis (Deep vein)	Sibling without cancer	54	<35	Multiple malignancies	≥ 5 years	NR	Not specified
Kero et al. (2023) [39]	Finland	Cardiac arrhythmia; cardiomyopathy; Cerebrovascular disease; Ischemic Heart Disease	Sibling without cancer	NR	<35	Acute lymphoblastic leukemia; acute myeloid leukemia	≥ 5 years	Median: 14.6 years (IQR 8.3-20.6)	Conventional chemotherapy; allogeneic hematopoietic stem cell transplantation
Khanna et al. (2019) [28]	Canada	Cardiac arrhythmia; CVD (overall); CAD; CHF; Pericardial disease; VHD	Matched general population	NR	<18	Multiple malignancies	≥5 years	Median: 10 years (range 0-25)	Anthracycline chemotherapy dose; chest-directed radiation
Marina et al. (2017) [29]	USA and Canada	Cardiac arrhythmia; CVD (overall); HF; HTN; MI; VHD	Sibling without cancer	52.7	<21	Ewing Sarcoma	≥5 years	NR	Anthracyclines, alkylating agents, radiation (including chest radiation), surgery
Mueller et al. (2013) [41]	USA and Canada	Stroke	Sibling without cancer	52	<21	Multiple malignancies	≥ 5 years	Median: 23.3 years	Cranial radiation; neck radiation; chemotherapy (anthracycline exposure evaluated)

Mulrooney et al. (2009) [34]	USA	HF; MI; Pericardial disease; VHD	Sibling without cancer	53.7	<21	Multiple malignancies	≥ 5 years	Median: 13 years (range 0-27)	Anthracycline chemotherapy dose; cardiac radiation dose; other chemotherapy agents including cyclophosphamide; cisplatin; bleomycin
Ocier et al. (2021) [30]	USA	Angina-chest pain; Cardiac arrhythmia; Conduction disorders; CVD (overall); Cardiomyopathy; HF; VHD; Pulmonary heart disease	Matched general population	54.6	< 65	Non-Hodgkin lymphoma	≥ 5 years	NR	Chemotherapy; radiation therapy; chemotherapy plus radiation; hematopoietic cell transplantation
Olsen et al. (2014) [31]	Denmark	Cardiac arrhythmia; Cardiomyopathy; CVD (overall); HF; Ischemic heart disease; Pericardial disease; Stroke; Stroke (hemorrhage); Thrombosis (Deep vein); VHD	Matched general population	57	< 18	Multiple malignancies	Not specified	Median: 27 years	Not specified
Pedersen et al. (2021) [13]	Denmark, Finland, Iceland, Sweden	Circulatory system disease	Matched general population	54.5	<20	Osteosarcoma and Ewing Sarcoma	≥5 years	Median: 12.5 years (range 0–42)	Not specified

Punyko et al. (2005) [35]	USA and Canada	Angina-like symptoms; Cardiac arrhythmia; HF; HTN; MI; Stroke	Sibling without cancer	57	<21	Rhabdomyosarcoma	≥5 years	Median: 15.7 years (range 5.2-28.8)	Surgery; chemotherapy; radiation therapy; combined modality therapy; specific agents including anthracyclines
Reulen et al. (2021) [14]	United Kingdom	Cerebrovascular disease	General population	54.6	<14	Multiple malignancies	≥ 5 years	Median: 15.8 years	Radiotherapy exposure classified including cranial radiotherapy, head and neck radiotherapy, and no cranial radiotherapy
Rugbjerg et al. (2014) [32]	Denmark	Cardiac arrhythmia; Cardiomyopathy; CVD (overall); Cerebrovascular disease; VHD; HF; HTN; Ischemic heart disease; Pulmonary heart disease; Venous and lymphatic disease; Arterial disease; Endo-, Peri-, Myocarditis	Matched general population	39.8	15–39	Multiple malignancies	≥ 1 years	Mean: 15 years (range 0-34)	Not specified
Termuhlen et al. (2011) [36]	USA and Canada	HF; HTN	Sibling without cancer	47	<21	Wilms tumor	≥5 years	NR	Radiation therapy; anthracyclines
Trama et al. (2022) [15]	Italy	Circulatory system disease	Matched general population	54	15–39	Leukemias and lymphomas	≥5 years	Median: 13 years (IQR 11–15)	Not specified

Van Den Belt-Dusebout et al. (2006) [16]	Netherlands	Angina pectoris; Cerebrovascular disease; CAD; HF; MI	Matched general population	100	NR	Testicular cancer	≥5 years	Median: 18.4 years (range: 5.0-38.4)	Surgery; radiotherapy (mediastinal, infradiaphragmatic); chemotherapy (PVB, BEP)
Weil et al. (2023) [37]	USA and Canada	HF	Sibling without cancer	45.5	<21	Wilms tumor	≥5 years	NR	Nephrectomy + VA; Nephrectomy + VAD + ART; Nephrectomy + VAD + ART + WLRT; Nephrectomy + ≥4 chemotherapy agents + any RT

Table S4. Baseline study characteristics for mortality [1,2,4,12,29,37,44–48,50–64].

Study	Country	Outcome(s)	Comparator Type	Male %	Age Range at Diagnosis (years)	Cancer Type (specific site if reported)	Time Since Diagnosis	Follow-up duration (median/mean; range; person-years if available)	Cancer Treatment Exposure
Anderson et al. (2019) [44]	USA	CVD Overall; Cerebrovascular disease; Aortic Aneurysm; Atherosclerosis; HTN without heart disease	Matched general population	60	15-39	Multiple malignancies	≥2 months	3.1 million person-years	Not reported
Armstrong et al. (2009) [45]	USA	CVD Overall	General population	NR	<21	Multiple malignancies	≥5 years	337,334 person-years	Radiotherapy; alkylating agents; anthracyclines; epipodophyllotoxins; bleomycin

Bagnasco et al (2019) [46]	Italy	CVD Overall	General population	56	<21	Multiple malignancies	≥5 years	Median: 22 years (IQR 17–29)	Chemotherapy; radiotherapy; combined chemotherapy and radiotherapy; surgery only
Bhandari et al. (2025) [47]	USA and Canada	CVD Overall	General population	54.5	<21	Multiple malignancies	≥5 years	Median: 43.5 years	Anthracyclines; alkylating agents; platinum agents; epipodophyllotoxins; radiation therapy (cranial, chest, abdominal); surgery; hematopoietic cell transplantation
Bottinor et al. (2024) [4]	USA and Canada	CAD; HF; Stroke	Sibling without cancer	53.6	<21	Multiple Malignancies	≥5 years	NR	Chemotherapy, radiation
Brewster et al. (2013) [58]	Scotland	Circulatory disease	General population	52.8	<25	All malignancies	≥ 5 years	58,358 person-years	Not reported
Castellino et al. (2011) [48]	USA and Canada	Cardiac ischemia; Cardiomyopathy; CVD Overall; Cerebrovascular disease	General population	57	<21	Hodgkin lymphoma	≥5 years	Median: 23.8 years (range 16-33)	Chemotherapy (included anthracyclines) only; radiotherapy only; chemotherapy plus radiotherapy
Fidler et al. (2016) [59]	United Kingdom	Circulatory disease; Cardiac ischemia; Cardiac arrhythmia; Cardiomyopathy; Pericardial disease; VHD	General population	54.9	<15	All malignancies	≥ 5 years	620,753 person-years	Not reported

Fosså et al. (2004) [60]	Norway	MI; Circulatory system disease	Matched general population	100	<55	Malignant germ cell tumors (predominantly testicular cancer)	Not specified	41,960 person years	Radiotherapy predominant in earliest era; combination radiotherapy and noncisplatin chemotherapy in intermediate era; cisplatin based chemotherapy and surgery in later era
Hancock et al. (1993) [12]	USA	CVD Overall; MI	General population	55.3	<21	Hodgkin lymphoma	Not specified	Mean: 10.3 years	Mediastinal radiation (dose-stratified); chemotherapy regimens (MOPP, ABVD, PAV, combinations); surgery
Henson et al. (2016) [1]	United Kingdom	Cardiac ischemia; Cardiomyopathy; VHD; CVD Overall	General population	38.2	15-39	Multiple malignancies	≥5 years	Mean: 14.3 years	Not reported
Kero et al. (2015) [50]	Finland	Cardiac ischemia/MI; CVD Overall	General population	44	<35	Multiple malignancies	≥ 5 years	NR	Not reported
Laverdière et al. (2009) [51]	USA and Canada	CVD Overall	General population	48	<21	Neuroblastoma	≥5 years	Median: 17.5 years	Surgery, chemotherapy, radiotherapy, multimodality therapy
MacArthur et al. (2007) [61]	Canada	Circulatory system disease	Matched general population	52	<20	Multiple malignancies	≥5 years	Mean: 15 (SD 7.2) years	Not reported

Marina et al. (2017) [29]	USA and Canada	CVD Overall	Sibling without cancer	52.7	<21	Ewing Sarcoma	≥5 years	NR	Anthracyclines, alkylating agents, radiation (including chest radiation), surgery
McKenzie et al. (2025) [62]	Canada	Circulatory system disease	Matched general population	54	<15	Multiple malignancies	≥5 years	Median: 9.1 years	Not reported
Mertens et al. (2001) [53]	USA	CVD Overall	General population	55.3	<21	Multiple malignancies	≥ 5 years	208,947 person-years	Radiation; alkylating agents; anthracyclines; epipodophyllotoxins; bleomycin
Mertens et al. (2008) [52]	USA and Canada	CVD Overall	General population	55.3	<21	Multiple malignancies	≥5 years	NR	Radiotherapy (including chest/spine for cardiac outcomes); anthracycline dose; alkylating agents; epipodophyllotoxins; bleomycin
Merzenich et al. (2022) [54]	Germany	CVD Overall	General population	55.5	<15	Multiple malignancies	≥5 years	Median: 21.7 years (range 0.0-29.97)	Not reported
Möller et al. (2001) [55]	Nordic countries (Denmark, Finland, Iceland, Norway, and Sweden)	CVD Overall	General population	52.2	<20	Multiple malignancies	≥ 5 years	Median: 14.9 years (range 5.0-35.9)	Not reported

Prasad et al. (2012) [63]	Finland	Circulatory system disease	General population	45.6	<35	Multiple malignancies	≥5 years	Mean: 15.9 years (range 0-38 years)	Not reported
Reulen et al. (2010) [56]	United Kingdom	CVD Overall; Cerebrovascular disease; Circulatory system disease	General population	NR	<15	Multiple malignancies	≥5 years	Median: 24.3 years (range 5-66)	Radiotherapy and chemotherapy
Schindler et al. (2016) [57]	Switzerland	CVD Overall	Matched general population	55.9	<15	Multiple malignancies	≥5 years	Mean: 17.5 years (range 5.0-36.9)	Radiotherapy; chemotherapy; hematopoietic stem cell transplantation
Wang et al. (2021) [2]	USA	CVD Overall; Cerebrovascular disease	Matched general population	38.1	15–39	Multiple malignancies	≥5 years	Mean: 13.9 years (range 0-36.9)	Not reported
Weil et al. (2023) [37]	USA and Canada	CVD Overall	Sibling without cancer	45.5	<21	Wilms tumor	≥5 years	NR	Nephrectomy + VA; Nephrectomy + VAD + ART; Nephrectomy + VAD + ART + WLRT; Nephrectomy + ≥4 chemotherapy agents + any RT
Youlden et al. (2021) [64]	Australia	Circulatory system disease	Matched general population	54.9	<15	Multiple malignancies	≥5 years	Median: 11.6 years (IQR 5.1-19.1)	Surgery, chemotherapy, radiotherapy, and transplant

Table S5. Multi-publication cohort sources contributing to the meta-analysis [4,14,18,22,23,25,29,31–41,45,47,49,50,52,53,56,59,70].

Cohort source	Studies, n	Contributing publications

Childhood Cancer Survivor Study (CCSS), USA/Canada	14	Armstrong 2009[45]; Mertens 2001[53]; Mertens 2008[52]; Mulrooney 2009[34]; Termuhlen 2011[36]; Mueller 2013[41]; Ginsberg 2010[25]; Marina 2017[29]; Punyko 2005[35]; Gurney 2003[40]; Weil 2023[37]; Bhandari 2025[47]; Bottinor 2024[4]; Becktell 2024 [18]
British Childhood Cancer Survivor Study (BCCSS), UK	5	Reulen 2010[56]; Reulen 2021[14]; Fidler 2016[59]; Fidler 2017[49]; Bright 2017[70]
Finnish Cancer Registry, Finland	3	Kero 2014[38]; Kero 2015[50]; Kero 2023[39] (potential within-cohort overlap between Kero 2014[38] and 2015[50])
Korean National Health Insurance Service (NHIS) Database	3	Choi 2024 (gastric cancer)[22]; Choi 2025 (cervical cancer)[23]; Jeon 2025[33] — distinct cancer subsets drawn from the same registry
Kaiser Permanente AYA Cancer Survivors Cohort, USA	3	Choi 2024[22] (gastric cancer); Choi 2025[23] (cervical cancer); Jeon 2025[33]
Danish Cancer Registry, Denmark	2	Olsen 2014[31]; Rugbjerg 2014[32]
Subtotal (multi-publication cohorts)	***30***	
Single-publication cohorts (distinct national/regional registries or institutional cohorts)	*28*	
**Total studies included**	**58**	

Abbreviations: AYA, adolescents and young adults; BCCSS, British Childhood Cancer Survivor Study; CCSS, Childhood Cancer Survivor Study; NHIS, National Health Insurance Service.

Cohort family assignments are based on the underlying registry or longitudinal cohort identified in each publication. Publications drawing from the same cohort report different cardiovascular outcomes, disease-specific subsets, or follow-up windows; each was retained for its respective outcome. Cohort overlap is most concentrated within the cardiovascular mortality pool, where multiple CCSS publications contribute distinct mortality endpoints, and this should be considered when interpreting the apparent precision of pooled mortality estimates.

Table S6. Reconciliation of unique studies and person-outcome totals contributing to each outcome [1,2,11–14,16–64,70].

Section 1. Cardiovascular Conditions

Study	Effect	Cancer cohort, n	Comparator cohort, n	Total, n
**Overall Cardiovascular Disease: 16 pooled studies (6 HR + 5 IRR + 5 RR)**				
Ajaj et al. (2025) [17]	HR	582	2,833	3,415
Olsen et al. (2014) [31]	HR	2,243	External§	2,243
Choi et al. (2025) [23]	HR	7,803	23,327	31,130
Khanna et al. (2019) [28]	HR	7,289	36,205	43,494
Ocier et al. (2021) [30]	HR	1,279	5,759	7,038
Choi et al. (2024) [22]	HR	6,562	19,678	26,240
Chao, Xu, & Bhatia et al. (2016) [21]	IRR	5,673	57,617	63,290
Chao et al. (2020) [19]	IRR	6,778	87,737	94,515
Hayek et al. (2023) [26]	IRR	12,674	50,696	63,370
Chao, Xu, & Bell et al. (2016) [20]	IRR	652	6,520	7,172
Keegan et al. (2025) [27]	IRR	14,917	149,164	164,081
Ginsberg et al. (2010) [25]	RR	403	3,899	4,302
Marina et al. (2017) [29]	RR	970	4,022	4,992
Faber et al. (2018) [24]	RR	951	External§	951
Rugbjerg et al. (2014) [32]	RR	43,153	255,513	298,666
Becktell et al. (2024) [18]	RR	1,257	5,051	6,308
Subtotal (16 unique studies)		**113,186**	**708,021**	**821,207**
**Cardiomyopathy: 9 pooled studies (6 HR + 3 IRR) + 1 non-pooled studies (1 RR)**				
Choi et al. (2025) [23]	HR	7,803	23,327	31,130
Ocier et al. (2021) [30]	HR	1,279	5,759	7,038
Olsen et al. (2014) [31]	HR	2,243	External§	2,243

Kero et al. (2014) [38]	HR	13,860	43,392	57,252
Kero et al. (2023) [39]	HR	1,370	39,217	40,587
Jeon et al. (2025) [33]	HR	4,553	13,636	18,189
Chao et al. (2020) [19]	IRR	6,778	87,737	94,515
Keegan et al. (2025) [27]	IRR	14,917	149,164	164,081
Hayek et al. (2023) [26]	IRR	12,674	50,696	63,370
Rugbjerg et al (2023) [32]	RR	43,153	255,513	298,666
Subtotal (10 unique studies)		**108,630**	**668,441**	**777,071**
**Heart Failure: 14 pooled studies (9 HR + 5 RR) + 1 non-pooled study (1 SIR)**				
Ajaj et al. (2025) [17]	HR	582	2,833	3,415
Choi et al. (2024) [22]	HR	6,562	19,678	26,240
Choi et al. (2025) [23]	HR	7,803	23,327	31,130
Ocier et al. (2021) [30]	HR	1,279	5,759	7,038
Olsen et al. (2014) [31]	HR	2,243	External§	2,243
Jeon et al. (2025) [33]	HR	4,553	13,636	18,189
Mulrooney et al. (2009) [34]	HR	14,358	3,899	18,257
Khanna et al. (2019) [28]	HR	7,289	36,205	43,494
Termuhlen et al. (2011) [36]	HR	1,256	4,023	5,279
Rugbjerg et al. (2014) [32]	RR	43,153	255,513	298,666
Faber et al. (2018) [24]	RR	951	External§	951
Weil et al. (2023) [37]	RR	2,008	5,006	7,014
Marina et al. (2017) [29]	RR	970	4,022	4,992
Punyko et al. (2005) [35]	RR	606	3,701	4,307
Van Den Belt-Dusebout et al. (2006) [16]	SIR	2,339	External§	2,339

Subtotal (15 unique studies)		**95,952**	**377,602**	**473,554**
**Cardiac Arrhythmia: 13 pooled studies (8 HR + 5 RR) + 1 non-pooled study (1 IRR)**				
Olsen et al. (2014) [31]	HR	2,243	External§	2,243
Choi et al. (2025) [23]	HR	7,803	23,327	31,130
Choi et al. (2024) [22]	HR	6,562	19,678	26,240
Kero et al. (2014) [38]	HR	13,860	43,392	57,252
Kero et al. (2023) [39]	HR	1,370	39,217	40,587
Ocier et al. (2021) [30]	HR	1,279	5,759	7,038
Khanna et al. (2019) [28]	HR	7,289	36,205	43,494
Ajaj et al. (2025) [17]	HR	582	2,833	3,415
Gurney et al. (2003) [40]	RR	1,607	3,418	5,025
Rugbjerg et al. (2014) [32]	RR	43,153	255,513	298,666
Punyko et al. (2005) [35]	RR	606	3,701	4,307
Ginsberg et al. (2010) [25]	RR	403	3,899	4,302
Marina et al. (2017) [29]	RR	970	4,022	4,992
Keegan et al. (2025) [27]	IRR	14,917	149,164	164,081
Subtotal (14 unique studies)		**102,644**	**590,128**	**692,772**
**Ischemic Heart Disease: 4 pooled studies (4 HR) + 2 non-pooled studies (1 IRR + RR)**				
Olsen et al. (2014) [31]	HR	2,243	External§	2,243
Choi et al. (2025) [23]	HR	7,803	23,327	31,130
Choi et al. (2024) [22]	HR	6,562	19,678	26,240
Kero et al. (2023) [39]	HR	1,370	39,217	40,587
Hayek et al. (2023) [26]	IRR	12,674	50,696	63,370
Rubjerg et al. (2014) [32]	RR	43,153	255,513	298,666
Subtotal (6 unique studies)		**73,805**	**388,431**	**462,236**

**Myocardial Infarction: 6 pooled studies (6 HR) + 3 non-pooled studies (1 SIR + 2 RR)**				
Choi et al. (2024) [22]	HR	6,562	19,678	26,240
Jeon et al. (2025) [33]	HR	4,553	13,636	18,189
Choi et al. (2025) [23]	HR	7,803	23,327	31,130
Ajaj et al. (2025) [17]	HR	582	2,833	3,415
Kero et al. (2014) [38]	HR	13,860	43,392	57,252
Mulrooney et al. (2009) [34]	HR	14,358	3,899	18,257
Van Den Belt-Dusebout et al. (2006) [16]	SIR	2,339	External§	2,339
Punyko et al. (2005) [35]	RR	606	3,701	4,307
Marina et al. (2017) [29]	RR	970	4,022	4,992
Subtotal (9 unique studies)		**51,633**	**114,488**	**166,121**
**Valvular Heart Disease: 5 pooled studies (5 HR) + 2 non-pooled studies (1 IRR + 1 RR)**				
Choi et al. (2025) [23]	HR	7,803	23,327	31,130
Ocier et al. (2021) [30]	HR	1,279	5,759	7,038
Olsen et al. (2014) [31]	HR	2,243	External§	2,243
Khanna et al. (2019) [28]	HR	7,289	36,205	43,494
Mulrooney et al. (2009) [34]	HR	14,358	3,899	18,257
Keegan et al. (2025) [27]	IRR	14,917	149,164	164,081
Marina et al. (2017) [29]	RR	970	4,022	4,992
Subtotal (7 unique studies)		**48,859**	**222,376**	**271,235**
**Pericardial Disease: 4 pooled studies (4 HR) + 1 non-pooled study (1 IRR)**				
Ajaj et al. (2025) [17]	HR	582	2,833	3,415
Khanna et al. (2019) [28]	HR	7,289	36,205	43,494
Olsen et al. (2014) [31]	HR	2,243	External§	2,243

Mulrooney et al. (2009) [34]	HR	14,358	3,899	18,257
Keegan et al. (2025) [27]	IRR	14,917	149,164	164,081
Subtotal (5 unique studies)		**39,389**	**192,101**	**231,490**
**Stroke: 11 pooled studies (5 HR + 3 IRR + 3 RR) 2 non-pooled studies (2 SHR)**				
Choi et al. (2024) [22]	HR	6,562	19,678	26,240
Choi et al. (2025) [23]	HR	7,803	23,327	31,130
Jeon et al. (2025) [33]	HR	4,553	13,636	18,189
Olsen et al. (2014) [31]	HR	2,243	External§	2,243
Ajaj et al. (2025) [17]	HR	582	2,833	3,415
Keegan et al. (2025) [27]	IRR	14,917	149,164	164,081
Hayek et al. (2023) [26]	IRR	12,674	50,696	63,370
Chao et al. (2020) [19]	IRR	6,778	87,737	94,515
Punyko et al. (2005) [35]	RR	606	3,701	4,307
Mueller et al. (2013) [41]	RR	14,358	4,023	18,381
Gurney et al. (2003) [40]	RR	1,607	3,418	5,025
Bright et al. (2017)* [70]	SHR	178,926	External§	178,926
Subtotal (12 unique studies)		**251,609**	**358,213**	**609,822**
**Cerebrovascular Disease: 4 pooled studies (4 HR) + 5 non-pooled studies (2 SHR + 1 SIR + 1 IRR + 1 RR)**				
Choi et al. (2024) [22]	HR	6,562	19,678	26,240
Choi et al. (2025) [23]	HR	7,803	23,327	31,130
Kero et al. (2023) [39]	HR	1,370	39,217	40,587
Gunn et al. (2015) [42]	HR	740	3,615	4,355
Bright et al. (2017) [70]	SHR	178,926	External§	178,926
Reulen et al. (2021) [14]	SHR	13,457	External§	13,457

Van Den Belt-Dusebout et al. (2006) [16]	SIR	2,339	External§	2,339
Chao, Xu, & Bell et al. (2016) [20]	IRR	652	6,520	7,172
Rugbjerg et al. (2014) [32]	IRR	43,153	255,513	298,666
Subtotal (9 unique studies)	RR	**255,002**	**347,870**	**602,872**
**Pulmonary Embolism: 3 pooled studies (3 HR)**				
Jeon et al. (2025) [33]	HR	4,553	13,636	18,189
Choi et al. (2025) [23]	HR	7,803	23,327	31,130
Choi et al. (2024) [22]	HR	6,562	19,678	26,240
Subtotal (3 unique studies)		**18,918**	**56,641**	**75,559**
**Venous Thrombosis: 5 pooled studies (5 HR) + 1 non-pooled study (1 RR)**				
Kero et al. (2014) [38]	HR	13,860	43,392	57,252
Choi et al. (2024) [22]	HR	6,562	19,678	26,240
Jeon et al. (2025) [33]	HR	4,553	13,636	18,189
Olsen et al. (2014) [31]	HR	2,243	External§	2,243
Choi et al. (2025) [23]	HR	7,803	23,327	31,130
Gurney et al. (2003) [40]	RR	1,607	3,418	5,025
Subtotal (6 unique studies)		**36,628**	**103,451**	**140,079**
**Hypertension: 7 pooled studies (3 IRR + 4 RR) + 2 non-pooled studies (2 HR)**				
Chao, Xu, & Bell et al. (2016) [20]	IRR	652	6,520	7,172
Hayek et al. (2023) [26]	IRR	12,674	50,696	63,370
Chao et al. (2020) [19]	IRR	6,778	87,737	94,515
Rugbjerg et al. (2014) [32]	RR	43,153	255,513	298,666
Marina et al. (2017) [29]	RR	970	4,022	4,992
Punyko et al. (2005) [35]	RR	606	3,701	4,307

Kantor et al. (1989) [43]	RR	119	External§	119
Ajaj et al. (2025) [17]	HR	582	2,833	3,415
Termuhlen et al. (2011) [36]	HR	1,256	4,023	5,279
Subtotal (9 unique studies)		**66,790**	**415,045**	**481,835**
**Circulatory System Disease: 4 non-pooled studies (1 HR + 1 SHR + 2 RR)**				
Gunn et al. (2015) [42]	HR	740	3,615	4,355
Trama et al. (2022) [35]	SHR	5,042	External§	5,042
Bhuller et al. (2016) [11]	RR	442	2,810	3,252
Pedersen et al. (2021) [13]	RR	620	3,049	3,669
Subtotal (4 unique studies)		**6,844**	**9,474**	**16,318**
**Coronary Artery Disease: 5 non-pooled studies (2 HR + 2 IRR + 1 SIR)**				
Ajaj et al. (2025) [17]	HR	582	2,833	3,415
Khanna et al. (2019) [28]	HR	7,289	36,205	43,494
Chao et al. (2020) [19]	IRR	6,778	87,737	94,515
Keegan et al. (2025) [27]	IRR	14,917	149,164	164,081
Van Den Belt-Dusebout et al. (2006) [16]	SIR	2,339	External§	2,339
Subtotal (5 unique studies)		**31,9055**	**275,9391**	**307,844**
**Angina/Chest Pain: 4 non-pooled studies (2 RR + 1 SIR + 1 HR)**				
Gurney et al. (2003) [40]	RR	1,607	3,418	5,025
Punyko et al. (2005) [35]	RR	606	3,701	4,307
Van Den Belt-Dusebout et al. (2006) [16]	SIR	2,339	External§	2,339
Ocier et al. (2021) [30]	HR	1,279	5,759	7,038
Subtotal (4 unique studies)		**5,831**	**12,878**	**18,709**

Section 2. Cardiovascular Mortality

Study	Effect	Cancer cohort, n	Comparator cohort, n	Total, n
**Overall Cardiovascular Mortality: 18 pooled studies (18 SMR) + 1 non-pooled study (1 RR)**				
Mertens et al. (2001) [53]	SMR	—	General population‡	20,227
Reulen et al. (2010) [56]	SMR	—	General population‡	17,981
Weil et al. (2023) [37]	SMR	—	5,006	7,014
Marina et al. (2017) [29]	SMR	—	4,022	4,992
Fidler et al. (2017) [49]	SMR	—	General population‡	34,489
Kero et al. (2015) [50]	SMR	—	43,892	60,661
Bhandari et al. (2025) [47]	SMR	—	General population‡	7,490
Castellino et al. (2011) [48]	SMR	—	General population‡	2,634
Armstrong et al. (2009) [45]	SMR	—	General population‡	20,483
Bagnasco et al. (2019) [46]	SMR	—	General population‡	12,214
Mertens et al. (2008) [52]	SMR	—	General population‡	20,483
Merzenich et al. (2022) [54]	SMR	—	General population‡	4,505
Schindler et al. (2016) [57]	SMR	—	General population‡	3,965
Möller et al. (2001) [55]	SMR	—	General population‡	13,711
Anderson et al. (2019) [44]	SMR	—	General population‡	235,641

Henson et al. (2016) [1]	SMR	—	General population‡	200,945
Laverdière et al. (2009) [51]	SMR	—	General population‡	1,358
Wang et al. (2021) [2]	SMR	—	General population‡	160,834
Hancock et al. (1993) [12]	RR	—	General population‡	635
Subtotal (19 unique studies)		**—**	**52,920**	**830,262**
**Circulatory System Disease Mortality: 8 pooled studies (8 SMR)**				
Brewster et al. (2013) [58]	SMR	—	General population‡	5,299
Fosså et al. (2004) [60]	SMR	—	General population‡	3,378
MacArthur et al. (2007) [61]	SMR	—	General population‡	2,354
McKenzie et al. (2025) [62]	SMR	—	General population‡	10,800
Reulen et al. (2010) [56]	SMR	—	General population‡	17,981
Youlden et al. (2021) [64]	SMR	—	General population‡	13,432
Fidler et al. (2016) [59]	SMR	—	General population‡	34,489
Prasad et al. (2012) [63]	SMR	—	General population‡	9,245
Subtotal (8 unique studies)		**—**	**—**	**96,978**
**Cardiac Ischemia Mortality: 4 pooled studies (4 SMR)**				
Castellino et al. (2011) [48]	SMR	—	General population‡	2,634
Fidler et al. (2017) [49]	SMR	—	General population‡	34,489

Henson et al. (2016) [1]	SMR	—	General population‡	200,945
Kero et al. (2015) [50]	SMR	—	43,892	60,661
Subtotal (4 unique studies)		**—**	**43,892**	**298,729**
**Cardiomyopathy Mortality: 3 pooled studies (3 SMR)**				
Castellino et al. (2011) [48]	SMR	—	General population‡	2,634
Fidler et al. (2017) [49]	SMR	—	General population‡	34,489
Henson et al. (2016) [1]	SMR	—	General population‡	200,945
Subtotal (3 unique studies)		**—**	**—**	**238,068**
**Cerebrovascular Disease Mortality: 4 pooled studies (4 SMR)**				
Reulen et al. (2010) [56]	SMR	—	General population‡	17,981
Wang et al. (2021) [2]	SMR	—	General population‡	160,834
Castellino et al. (2011) [48]	SMR	—	General population‡	2,634
Anderson et al. (2019) [44]	SMR	—	General population‡	235,641
Subtotal (4 unique studies)		**—**	**—**	**417,090**

Abbreviations: HR, hazard ratio; IRR, incidence rate ratio; RR, risk ratio; SMR, standardized mortality ratio.

Studies are listed once per pool to which they contribute; subtotals reflect unique studies.

* Bright et al. (2017) [70] contributed two stroke outcome estimates (ischemic and hemorrhagic stroke); the cohort is counted once to avoid double-counting.

§ **External comparator:** Studies using externally matched general-population reference cohorts whose denominators were not separately reported. For these studies, the cancer cohort count reflects the full study sample; comparator population is implicit.

‡ **General population:** Standardized mortality ratios are calculated against national or regional general-population reference rates rather than an enumerated comparator cohort; participant totals reflect the cancer cohort only.

Table S7. Results of non-pooled data [1,4,11–17,19,20,26–30,32,35,36,40,42,44,49,60,70].

Outcome	Study	effect estimate
**Conditions**		
Circulatory system disease	Gunn et al. (2015)[42]	HR 2.7 (1.5-5.0)
Circulatory system disease	Trama et al. (2022)[15]	SHR 1.5 (1.3–1.6)
Circulatory system disease	Bhuller et al. (2016)[11]	RR 1.42 (0.89–2.27)
Circulatory system disease	Pedersen et al. (2021)[13]	RR 2.6 (1.8–3.9)
Cardiomyopathy	Rugbjerg et al. (2014)[32]	RR 1.95 (1.67-2.26)
Heart failure	Van Den Belt-Dusebout et al. (2006)[16]	SIR 0.93 (0.71–1.20)
Cardiac arrhythmia	Keegan et al. (2025)[27]	IRR 1.96 (1.79-2.14)
Ischemic heart disease	Hayek et al. (2023)[26]	IRR 1.37 (1.13-1.65)
Ischemic heart disease	Rugbjerg et al. (2014)[32]	RR 1.23 (1.18 to 1.28)
Myocardial infarction	Van Den Belt-Dusebout et al. (2006)[16]	SIR 1.11 (0.94–1.31)
Myocardial infarction	Punyko et al. (2005)[35]	RR 6.0 (1.1–36.6)
Myocardial infarction	Marina et al. (2017)[29]	RR 5.0 (2.2–11.5)
Coronary artery disease	Ajaj et al. (2025)[17]	HR 1.8 (0.3–9.1)
Coronary artery disease	Khanna et al. (2019)[28]	HR 3.4 (1.5–7.7)
Coronary artery disease	Chao et al. (2020)[19]	IRR 1.63 (1.14–2.34)
Coronary artery disease	Keegan et al. (2025)[27]	IRR 1.75 (1.46-2.10)
Coronary artery disease	Van Den Belt-Dusebout et al. (2006)[16]	SIR 1.17 (1.04–1.31)
Valvular heart disease (VHD)	Keegan et al. (2025)[27]	IRR 4.54 (3.63-5.67)
Valvular heart disease (VHD)	Marina et al. (2017)[29]	RR 2.8 (1.6–5.2)
Pericardial disease	Keegan et al. (2025)[27]	IRR 5.47 (4.05-7.39)
Cerebrovascular disease	Bright et al.(2017)[70]	SHR 1.4 (1.3–1.4)
Cerebrovascular disease	Reulen et al. (2021)[14]	SHR 4.0 (3.7 to 4.3)
Cerebrovascular disease	Van Den Belt-Dusebout et al. (2006)[16]	SIR 0.84 (0.62–1.11)
Cerebrovascular disease	Chao, Xu, & Bell et al. (2016)[20]	IRR 10.0 (2.7–37.4)
Cerebrovascular disease	Rugbjerg et al. (2014)[32]	RR 1.33 (1.27 to 1.40)
Stroke	Bright et al.(2017)[70]	SHR 1.5 (1.4–1.6)
Stroke-cerebral hemorrhage	Bright et al.(2017)[70]	SHR 2.0 (1.8–2.1)
Thrombosis	Gurney et al. (2003)[40]	RR 5.7 (3.2–10.0)
Hypertension	Ajaj et al. (2025)[17]	HR 1.0 (0.6–1.5)
Hypertension	Termuhlen et al. (2011)[36]	HR 8.2 (6.4–10.5)
Angina/chest pain	Gurney et al. (2003)[40]	RR 2.0 (1.5–2.7)

Angina/chest pain	Punyko et al. (2005)[35]	RR 2.0 (1.3–2.9)
Angina/chest pain	Van Den Belt-Dusebout et al. (2006)[16]	SIR 1.20 (1.01–1.40)
Angina/chest pain	Ocier et al. (2021)[30]	HR 1.54 (1.10-2.18)
Conduction disorders	Ocier et al. (2021)[30]	HR 1.84 (1.04-3.26)
Endo-, peri-, myocarditis and valvular disease	Rugbjerg et al. (2014)[32]	RR 1.89 (1.75-2.05)
Hyperlipidemia	Hayek et al. (2023)[26]	IRR 1.33 (1.24-1.43)
Arterial disease	Rugbjerg et al. (2014)[32]	RR 1.55 (1.47-1.64)
Venous and lymphatic disease	Rugbjerg et al. (2014)[32]	RR 1.32 (1.27-1.37)
**Mortality**		
Overall CV	Hancock et al. (1993)[12]	RR 29.6 (16.0-49.3)
Heart failure	Bottinor et al. (2024)[4]	HR 3.62 (0.48–27.29)
Cardiac arrhythmia	Fidler et al. (2017)[49]	SMR 3.4 (1.1–8.0)
Myocardial infarction	Fosså et al. (2004)[60]	SMR 1.1 (0.9-1.4)
myocardial infarction	Hancock et al. (1993)[12]	RR 41.5 (18.1-82.1)
Coronary artery disease	Bottinor et al. (2024)[4]	HR 3.70 (1.05–13.02)
Valvular heart disease	Fidler et al. (2017)[49]	SMR 3.6 (1.6–7.2)
Valvular heart disease	Henson et al. (2016)[1]	SMR 2.9 (2.4 to 3.5)
Pericardial disease	Fidler et al. (2017)[49]	SMR 8.0 (1.0–29.0)
Stroke	Bottinor et al. (2024)[4]	HR 3.12 (0.40–24.32)
Aortic aneurysm and dissection	Anderson et al. (2019)[44]	SMR 1.01 (0.71-1.39)
Atherosclerosis	Anderson et al. (2019)[44]	SMR 1.95 (1.19–3.01)
Hypertension without heart disease	Anderson et al. (2019)[44]	SMR 1.32 (1.04–1.65)

Table S8. Sensitivity analysis results.

Outcome		Including all studies per cohort			Including one study per cohort			
outcome	Effect Estimate	Number of studies	Effect (95%CI)	P-value (of effect)	Change	Adjusted number of studies	Effect (95%CI)	P-value (of effect)
Conditions								
Cardiac arrhythmia	HR	8	1.3740 [1.1932 to 1.5821]	< 0.0001	1	7	1.3590 [1.1502; 1.6056]	0.0003
Cardiac arrhythmia	RR	5	1.4835 [0.9802 to 2.2450]	0.0621	3	2	0.9852 [0.5923; 1.6388]	0.9542

cardiomyopathy	HR	6	3.5230 [2.2054 to 5.6275]	< 0.0001	1	5	3.1332 [1.8223; 5.3871]	< 0.0001
Heart failure	HR	9	4.0808 [2.1986 to 7.5744]	< 0.0001	1	8	3.3574 [1.9782; 5.6984]	< 0.0001
Heart failure	RR	5	9.4103 [2.9217 to 30.3096]	0.0002	2	3	4.5835 [1.2242; 17.1614]	0.0238
Cardiovascular disease-overall	IRR	5	2.2016 [2.0522 to 2.3618]	< 0.0001	2	3	2.1499 [2.0305; 2.2764]	< 0.0001
Cardiovascular disease-overall	RR	5	2.3735 [1.4081 to 4.0009]	0.0012	2	3	2.0421 [1.1343; 3.6763]	0.0173
Hypertension	IRR	3	1.3414 [1.2484 to 1.4413]	< 0.0001	1	2	1.3431 [1.2495; 1.4436]	< 0.0001
Hypertension	RR	4	1.2906 [1.0987 to 1.5161]	0.0019	1	3	1.3445 [1.0568; 1.7106]	0.016
Mortality								
Cardiovascular mortality	SMR	18	4.2996 [3.0573 to 6.0468]	< 0.0001	7	11	4.4824 [2.7688; 7.2567]	< 0.0001
Cerebrovascular mortality	SMR	4	2.4392 [1.1683 to 5.0923]	0.0176	1	3	3.0995 [1.2962; 7.4118]	0.011
Circulatory disease mortality	SMR	8	3.2888 [2.1176 to 5.1077]	< 0.0001	1	7	3.2123 [1.9260; 5.3575]	< 0.0001

Table S9. Results of meta-regression analysis.

Moderators	Coefficient (95% CI, to UCI)	Std Error	p-value
**OUTCOME 1: CV MORTALITY**			

**Country**			
Finland (reference)			
Germany	0.9854 (-1.0220 to 2.9929)	1.0242	0.336
Italy	1.3994 (-0.5436 to 3.3424)	0.9913	0.1581
Nordic countries (Denmark, Finland, Iceland, Norway, and Sweden)	1.116 (-0.8341 to 3.0661)	0.995	0.262
Switzerland	1.8997 (-0.0885 to 3.8880)	1.0144	0.0611
United Kingdom	0.2929 (-1.2840 to 1.8699)	0.8046	0.7158
USA	0.5549 (-0.9720 to 2.0818)	0.7791	0.4763
USA and Canada	1.0992 (-0.3930 to 2.5915)	0.7614	0.1488
**Continuous moderators**			
Percentage of male	0.0672 (0.0217 to 0.1126)	0.0232	0.0038
